# Variation in NAT2 acetylation phenotypes is associated with differences in food-producing subsistence modes and ecoregions in Africa

**DOI:** 10.1186/s12862-015-0543-6

**Published:** 2015-12-01

**Authors:** Eliška Podgorná, Issa Diallo, Christelle Vangenot, Alicia Sanchez-Mazas, Audrey Sabbagh, Viktor Černý, Estella S. Poloni

**Affiliations:** Department of the Archaeology of Landscape and Archaeobiology, Archaeogenetics Laboratory, Institute of Archaeology of the Academy of Sciences of the Czech Republic, Prague, Czech Republic; Department of Genetics and Evolution, Anthropology Unit, Laboratory of Anthropology, Genetics and Peopling History, University of Geneva, 12 Rue Gustave-Revilliod, 1211 Geneva 4, Switzerland; Département de Linguistique et Langues Nationales, Institut des Sciences des Sociétés, CNRST, Ouagadougou, Burkina Faso; IRD, UMR216, Mère et enfant face aux infections tropicales, Université Paris Descartes, Sorbonne Paris Cité, Faculté des Sciences Pharmaceutiques et Biologiques, Paris, France

**Keywords:** NAT2, Acetylation polymorphism, African Sahel, Pastoral nomads, Subsistence mode, Ecoregion, Natural selection

## Abstract

**Background:**

Dietary changes associated to shifts in subsistence strategies during human evolution may have induced new selective pressures on phenotypes, as currently held for lactase persistence. Similar hypotheses exist for arylamine N-acetyltransferase 2 (NAT2) mediated acetylation capacity, a well-known pharmacogenetic trait with wide inter-individual variation explained by polymorphisms in the *NAT2* gene. The environmental causative factor (if any) driving its evolution is as yet unknown, but significant differences in prevalence of acetylation phenotypes are found between hunter-gatherer and food-producing populations, both in sub-Saharan Africa and worldwide, and between agriculturalists and pastoralists in Central Asia. These two subsistence strategies also prevail among sympatric populations of the African Sahel, but knowledge on *NAT2* variation among African pastoral nomads was up to now very scarce. Here we addressed the hypothesis of different selective pressures associated to the agriculturalist or pastoralist lifestyles having acted on the evolution of *NAT2* by sequencing the gene in 287 individuals from five pastoralist and one agriculturalist Sahelian populations.

**Results:**

We show that the significant *NAT2* genetic structure of African populations is mainly due to frequency differences of three major haplotypes, two of which are categorized as decreased function alleles (NAT2*5B and NAT2*6A), particularly common in populations living in arid environments, and one fast allele (NAT2*12A), more frequently detected in populations living in tropical humid environments. This genetic structure does associate more strongly with a classification of populations according to ecoregions than to subsistence strategies, mainly because most Sahelian and East African populations display little to no genetic differentiation between them, although both regions hold nomadic or semi-nomadic pastoralist and sedentary agriculturalist communities. Furthermore, we found significantly higher predicted proportions of slow acetylators in pastoralists than in agriculturalists, but also among food-producing populations living in the Sahelian and dry savanna zones than in those living in humid environments, irrespective of their mode of subsistence.

**Conclusion:**

Our results suggest a possible independent influence of both the dietary habits associated with subsistence modes and the chemical environment associated with climatic zones and biomes on the evolution of *NAT2* diversity in sub-Saharan African populations.

**Electronic supplementary material:**

The online version of this article (doi:10.1186/s12862-015-0543-6) contains supplementary material, which is available to authorized users.

## Background

All along human evolution and continuing in present times, populations have developed a wide variety of cultural innovations. Many of these may have affected the interactions between humans and their environment in such a way as to leave gene-culture coevolution imprints in the genome, due to both demographic and selective processes [1–5]. Among these innovations, new subsistence strategies represent major shifts with notable consequences on the pathogenic and dietary environments in which populations have been living, thus possibly also inducing new selective pressures on phenotypes [6–9]. Subsistence strategies are broadly classified in two opposite categories, i.e., foragers (or hunter-gatherers), relying on the collection of nutriments naturally occurring in the wild, and food-producers whose appearance in the prehistoric record is generally associated with the emergence of the Neolithic [10–12]. Among the latter, two major modes of production are distinguished, namely agriculture based on the cultivation of domesticated plants, and pastoralism based on the herding of domesticated animals. These two modes of subsistence also imply differential usage of space, in that most agricultural societies developed sedentarism whereas communities relying on animal husbandry developed nomadism or transhumance to exploit seasonal variations in the location of pasture areas.

It is currently held that these differential developments in subsistence strategies also influenced the selective regimes to which past populations were subjected, including through the action of new population-specific (i.e., culturally-related) selective pressures induced by dietary changes [13–15]. Lactase persistence, a heritable condition in which the physiological ability to digest lactose (the sugar contained in fresh milk) is maintained throughout adulthood, probably represents the best-known example of an adaptation related to diet [16]. Indeed, convergent evolution of the trait has been demonstrated [17], and is explained by the emergence of similar selective pressures resulting from adopting a diet heavily relying on milk. However, demographic processes (as opposed to selective pressure) playing a significant role in the spread of the trait constitutes a valid, mutually non-exclusive alternative explanation [18–22]. Hence, disentangling the respective contributions of selective and demographic forces to extant levels and patterns of genetic differentiation between populations represents a challenging task [23].

Reduced arylamine *N*-acetyltransferase 2 (NAT2) activity is another trait whose evolution was probably also shaped by differential, population-specific selective pressures [24–26]. Due to its early discovery linked to its major role in the treatment of tuberculosis with isoniazid, inherited variation in *N*-acetylation activity is currently one of the best known pharmacogenetic traits [27]. The phenotype is driven by the existence of a polymorphic *N*-acetyltransferase 2 (NAT2) enzyme. This cytosolic enzyme, encoded by the gene *NAT2* of chromosome 8, is mainly expressed in the liver, small intestine and colon [28] where it catalyzes a Phase II acetylation reaction, i.e., the transfer of an acetyl functional group to the terminal nitrogen of aromatic amines, heterocyclic amines, and hydrazines [29]. Hydrazines are used in the synthesis of numerous organic molecules, like the anti-tubercular agent isoniazid, while aromatic and heterocyclic amines are known to be produced, for instance, in meat and fish cooked at high temperatures, as well as in combustion smokes such as those from tobacco, grasses and wood chips [30, 31]. Thus, the NAT2 enzyme plays a crucial role in the detoxification of numerous xenobiotic compounds, including common therapeutic drugs and exogenous chemicals present in the diet and the environment [32]. Acetylation capacity (also coined acetylation status) can be measured by an individual’s response to drugs metabolized by the enzyme, and this phenotype is now known to depend on the individual’s genotype at the single coding exon of the *NAT2* gene. Mutations in *NAT2* result indeed in diverse acetylation phenotypes that explain inter-individual variation in response to standard drug dose administration (which can vary from lack of therapeutic efficacy to adverse drug reactions) [27, 33, 34]. Moreover, mutations in *NAT2* may also act as risk factors for different types of cancers [35, 36]. The gene displays a high degree of polymorphism in humans, with 88 alleles (i.e., haplotypes) listed to date by the official consensus gene nomenclature of human *NAT2* alleles (Arylamine *N*-acetyltransferase Gene Nomenclature Committee, nat.mbg.duth.gr, see [37]). Inter-population variation in *NAT2* allele frequencies has been intensively documented, particularly so for those alleles defined by the combination of nucleotides at four major functional single nucleotide polymorphisms (rs1801279, rs1801280, rs1799930, and rs1799931) of the coding exon [38] (and references therein).

Although response to drug intake is a quantitative variable, the distribution of the phenotype in tested human groups was shown to be at least bi- (or tri-) modal [39]. A simplified model of genotype-phenotype relationships was therefore adopted from the early studies onwards, in which those alleles considered as fully functional are responsible for the fast acetylator status, whereas decreased function alleles are responsible for the slow acetylator status [39–41]. Hence, acetylation status of an individual that is carrier of two fully functional alleles is classified as fast, that of a carrier of one fully functional allele and one allele with decreased function is classified as intermediate, and that of a carrier of two decreased function alleles as slow. Also, many studies do not distinguish between fast and intermediate acetylators, categorizing both types of subjects as fast (or rapid) acetylators. Similarly to lactase persistence, *NAT2* phenotypes prevalence varies substantially between populations [24–26, 38, 42–44], and hypotheses of gene-culture coevolution of *NAT2* polymorphisms have been put forwards, notably the idea that the slow acetylator phenotype became selectively advantageous in those populations adopting a food-producing mode of subsistence in the Neolithic. Indeed, given its role in the detoxification of numerous exogenic compounds, the NAT2 enzyme acts at the interface between the organism and its chemical environment, and hence is a likely target for natural selection. However, in contrast to lactose for lactase persistence, a dietary or environmental causal factor driving *NAT2* evolution is not identified, but hypotheses involving meat consumption and availability in food supplied folates related to nutritional shifts during the Neolithic have been proposed [25].

These hypotheses have been fuelled by the finding of significantly different frequency distributions of acetylation phenotypes between agriculturalists and hunter-gatherers, both in sub-Saharan Africa [44] and globally at the worldwide scale [25, 38, 45]. In addition, significant differential acetylation prevalence between sedentary agriculturalists and nomadic pastoralists has been reported in Central Asia [43]. Here, a higher proportion of slow acetylators was found among Tajik agriculturalists than among Kirghiz nomadic pastoralists. These two distinct food-producing strategies also prevail among sympatric populations of the African Sahel, but knowledge on *NAT2* genetic variation among African pastoral nomads was up to now very scarce, thus hindering a meaningful statistical analysis of the likely differences between food-producing subsistence modes in sub-Saharan Africa (see Supporting Information in [38]).

Current research holds that the earliest food-producing communities in sub-Saharan Africa relied on nomadic cattle herding, while sedentary farming would be a more recent phenomenon [46–50]. As the southern part of the African continent was occupied rather recently by Bantu herders, the most diversified pastoral societies live nowadays in the Sahelian belt, and these are, from west to east (and mentioning only the most important ones), the Moors, Fulani, Tuareg, Tubu, Zaghawa, nomadic Arabs (Kababish and Baggara) and Beja. This region forms a unique ecosystem bordered by the Saharan desert and the tropical rainforests, extending from east to west all across the continent. Its climate is characterized by annual cycles of wet and dry seasons allowing the co-existence of sedentary farmers and nomadic pastoralists who move with their livestock between wet- and dry-season pastures [51, 52]. In this study, we carried out an investigation of *NAT2* sequence variation among six Sahelian populations relying either on nomadic pastoralism or on sedentary agriculture sampled in an area extending from western Burkina Faso to northeastern Chad, in order to explicitly address the hypothesis that different selective pressures associated to these lifestyles acted on the evolution of *NAT2*. Moreover, by combining our dataset with published African samples of *NAT2* sequences (Table 1 and Additional file 1: Figure S1), we also address the hypothesis that selective pressures, stemming from an environmental factor linked to the ecoregion in which populations have been living (i.e., climatic zone and biome), might also have shaped the evolution of this gene.

**Table 1:** African population samples studied for *NAT2* sequence variation

		Sample	Geographical	Living in dry	Linguistic	Subsistence	
Population	Code	size	region (Country)	savanna biome^1^	affiliation	mode	Reference
Egyptians	EGY	10	Northern (Egypt)	no	Afro-Asiatic	Agricultural	[25]
Mandenka	MAN	97	Western (Senegal)	yes	Niger-Congo	Agricultural	[24]
Fulani Banfora	FBAN	49	Western (Burkina Faso)	yes	Niger-Congo	Pastoralist	this study
Fulani Tindangou	FTIN	50	Western (Burkina Faso)	yes	Niger-Congo	Pastoralist	this study
Fulani Ader	FADE	48	Western (Niger)	yes	Niger-Congo	Pastoralist	this study
Yoruba in Ibadan	YRI	88	Western (Nigeria)	no	Niger-Congo	Agricultural	[53]
Yoruba Bantus	YOR	31	Western (Nigeria)	no	Niger-Congo	Agricultural	[44]
Yoruba	YRB	18	Western (Nigeria)	no	Niger-Congo	Agricultural	[97]
Yoruba CEPH	YO	12	Western (Nigeria)	no	Niger-Congo	Agricultural	[42]
Ibo	IBO	19	Western (Nigeria)	no	Niger-Congo	Agricultural	[97]
Hausa	HAU	17	Western (Nigeria)	yes	Afro-Asiatic	Agricultural	[97]
Kanembou	KANE	49	Central (Chad)	yes	Nilo-Saharan	Agricultural	this study
Daza	DAZ	41	Central (Chad)	yes	Nilo-Saharan	Pastoralist	this study
Fulani Bongor	FBON	50	Central (Chad)	yes	Niger-Congo	Pastoralist	this study
Fulani	FU	13	Central (Cameroon)	yes	Niger-Congo	Pastoralist	[42]
Kanuri	KN	12	Central (Cameroon)	yes	Nilo-Saharan	Agricultural	[42]
Mada	MD	14	Central (Cameroon)	yes	Afro-Asiatic	Agricultural	[42]
Ngumba Bantus	NGU	16	Central (Cameroon)	no	Niger-Congo	Agricultural	[44]
Lemande	LM	14	Central (Cameroon)	no	Niger-Congo	Agricultural	[42]
Bakola Pygmy	PYG	26	Central (Cameroon)	no	Niger-Congo	Hunter-gatherer	[44]
Bedzan Pygmy	BEZ	32	Central (Cameroon)	no	Niger-Congo	Hunter-gatherer	[44]
Baka Pygmy Cameroon	BAKC	31	Central (Cameroon)	no	Niger-Congo	Hunter-gatherer	[44]
Baka Pygmy Gabon	BAKG	16	Central (Gabon)	no	Niger-Congo	Hunter-gatherer	[44]
Akele Bantus Gabon	GAB	26	Central (Gabon)	no	Niger-Congo	Agricultural	[44]
Biaka Pygmy	BIA	24	Central (C. A. R.)	no	Niger-Congo	Hunter-gatherer	[44]
Mbuti Pygmy	MBU	24	Central (D. R. C.)	no	Nilo-Saharan	Hunter-gatherer	[44]
Dinka	DN	13	Eastern (South Sudan)	yes	Nilo-Saharan	Pastoralist	[42]
Luhya in Webuye	LWK	97	Eastern (Kenya)	yes	Niger-Congo	Agricultural	[53]
Maasai	MAS	12	Eastern (Kenya)	yes	Nilo-Saharan	Pastoralist	[97]
Luo	LUO	14	Eastern (Kenya)	yes	Nilo-Saharan	Pastoralist	[97]
Somali	SOM	20	Eastern (Somalia)	yes	Afro-Asiatic	Pastoralist	[44]
Turu	TR	15	Eastern (Tanzania)	yes	Niger-Congo	Agro-pastoralist	[42]
Hadza	HZ	14	Eastern (Tanzania)	yes	Khoisan	Hunter-gatherer	[42]
Sandawe	SW	18	Eastern (Tanzania)	yes	Khoisan	Hunter-gatherer	[42]
Burunge	BG	17	Eastern (Tanzania)	yes	Afro-Asiatic	Agro-pastoralist	[42]
Chagga Bantus	CHA	32	Eastern (Tanzania)	yes	Niger-Congo	Agricultural	[44]
Maasai	MS	14	Eastern (Tanzania)	yes	Nilo-Saharan	Pastoralist	[42]
San	SAN	38	Eastern (Zimbabwe)	yes	Khoisan	Hunter-gatherer	[97]
African Americans^2^	ASW	61	ND^3^	ND^3^	ND^3^	ND^3^	[53]

^1^Classification according to climatic zone and biome (ecoregion), based on [99]

^2^African Americans: Americans of African ancestry in Southwestern US

^3^ND: not defined

## Results

### *NAT2* diversity in the Sahel

**Fig. 1:**
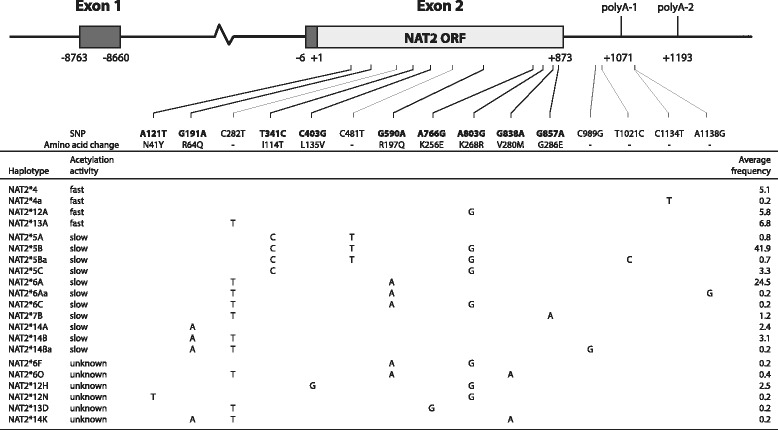
Schematic diagram of the *NAT2* locus on 8p22, including the non-coding 100 bp-long Exon 1, the 8.6 Kb-long intronic region, and the 870 bp-long single protein-coding Exon 2, adapted from [102] and [77]. The first (+1) and last positions (+873) of the open reading frame (ORF) of Exon 2 are indicated at the bottom of it, as well the relative positions of Exon 1 and of the polyadenylation signals. The positions of the 15 polymorphic sites (SNPs) observed among the 287 individuals from the six Sahelian samples sequenced in this study are shown as heavy-black (non-synonymous mutations) or light-gray (synonymous mutations) vertical bars below the diagram. Segments link the SNPs positions to the list of 21 haplotypes inferred from the combination of the 15 SNPs. Haplotypes’ associated acetylation activity (taken from the official NAT2 gene nomenclature, http://nat.mbg.duth.gr/) and average frequency among the six Sahelian samples are shown on the left and right sides of the list, respectively. A diagram displaying the position of the 30 SNPs observed in protein-coding Exon 2 among the 39 African population samples (1,192 individuals) analyzed in this study is shown in Additional file 3: Figure S2

We analyzed a total number of 287 samples from six well-defined Sahelian populations relying on two different modes of subsistence, namely pastoralism (Fulani from Banfora and from Tindangou in Burkina Faso, Fulani from Ader in Niger, Fulani from Bongor in Chad, and Daza in Chad) and agriculture (Kanembou in Chad). We completed this new dataset of *NAT2* sequences with those from other African population published samples (Table 1 and Additional file 1: Figure S1). For each of the 287 Sahelian samples, 1,396 base pairs (bp) encompassing the 870 bp *NAT2* coding exon were successfully sequenced (Additional file 2). In total, 15 polymorphic positions were observed, 11 of which are located in the coding exon, and 9 being non-synonymous (Fig. 1).

From the 15 single nucleotide polymorphisms (SNPs) observed, a total of 21 haplotypes were inferred with the expectation-maximisation (EM) algorithm of Arlequin for the six Sahelian populations (Fig. 1, Table 2 and Additional file 2). Among these, four haplotypes differed from known haplotypes at positions outside the coding exon, and were named with alphabetical suffixes (NAT2*4a, NAT2*5Ba, NAT2*6Aa, and NAT2*14Ba), while their predicted effect on the enzyme’s activity was considered unchanged from the defining haplotype (respectively, NAT2*4, NAT2*5B, NAT2*6A, and NAT2*14B). The EM algorithm also led to the inference of three new haplotypes, and the following names were attributed by the official *NAT2* nomenclature committee (nat.mbg.duth.gr): NAT2*12N, NAT2*13D and NAT2*14K. All three are defined by a new combination of recognized signature SNPs from the official *NAT2* gene nomenclature with other SNPs, so that enzymatic activity could not be predicted for any of these three haplotypes.

**Table 2:** Haplotype frequencies and molecular diversity of the six Sahelian samples in a 1,396 bp sequence encompassing the *NAT2* coding exon

		Population^1^						
Haplotype	Acetylation activity^2^	FBAN	FTIN	FADE	FBON	DAZ	KANE	Total
NAT2*4	fast	2	4	6	8	7	2	29
NAT2*4a	fast						1	1
NAT2*12A	fast	6	5		4	11	6	32
NAT2*13A	fast	11	10	8	7	2	2	40
NAT2*5A	slow		1		1		3	5
NAT2*5B	slow	39	46	42	47	28	40	242
NAT2*5Ba ^3^	slow			2	1		1	4
NAT2*5C	slow	3	4	6	2	1	3	19
NAT2*6A	slow	32	17	20	23	23	25	140
NAT2*6Aa ^3^	slow					1		1
NAT2*6C	slow				1			1
NAT2*7B	slow		1		2	2	2	7
NAT2*14A	slow	2	4	7		1		14
NAT2*14B	slow	3	5	2	2	1	5	18
NAT2*14Ba ^3^	slow			1				1
NAT2*6F	unknown		1					1
NAT2*6O	unknown					1	1	2
NAT2*12H	unknown			2	1	4	7	14
NAT2*12N ^4^	unknown		1					1
NAT2*13D ^4^	unknown				1			1
NAT2*14K ^4^	unknown		1					1
Total (2N chromosomes)		98	100	96	100	82	98	574
Number of haplotypes (k)		8	13	10	13	12	13	
Number of segregating sites (S)		6	9	9	10	10	11	
Gene diversity (expected heterozygosity, h)		0.72	0.75	0.75	0.72	0.78	0.76	
Nucleotide diversity (π) x 10^−3^		1.81	1.79	1.85	1.78	1.83	1.93	
Tajima’s *D* (*P*-value)^5^		**2.65 (0.994)**	1.09 (0.875)	1.18 (0.891)	0.73 (0.801)	0.72 (0.799)	0.68 (0.875)	

^1^Population codes as in Table 1

^2^Reported activity in the official *NAT2* gene nomenclature (nat.mbg.duth.gr)

^3^Small caps alphabetical suffixes were added to the names of haplotypes that differ from known haplotypes in the flanking region of the *NAT2* coding exon (see text)

^4^New haplotypes submitted to the official *NAT2* gene nomenclature and included in it (see text)

^5^ *P*-value associated with Tajima’s *D* test for departure from selective neutrality: it is given as the proportion of random *D* values generated under the neutral equilibrium model that are smaller than, or equal to the observed value. The sole significant result is shown in bold; it corresponds to a type I error rate of 0.006, and it remains significant after Bonferroni correction for multiple testing

Frequency distributions of the 21 *NAT2* haplotypes are reported in Table 2. Low-activity haplotype NAT2*5B is the most common haplotype in all six populations (41.9 % on average, Fig. 1). It is followed by NAT2*6A (24.5 % on average), also a low-activity haplotype. Thus, these two slow haplotypes together account for 67.4 % of the gene copies in the total sample of our study. Further low-activity haplotypes detected in most of our samples include NAT2*5C, *14A, and *14B, as well as *5A, *7B and *6C*. In turn, only three different fast haplotypes were detected in the Sahelian samples (NAT2*4, NAT2*12A and NAT2*13A), and these represent on average less than 18 % of the gene copies. Finally, six haplotypes could not be classified according to their effect on acetylation status. Their total frequency is of 3 % or less in the Fulani samples, whereas it is of 8.2 % and 6.1 % in the Kanembou and Daza, respectively.

Thus, when considering the whole 1,396 bp sequenced segment, 6 to 11 segregating sites, and 8 to 13 haplotypes were observed in each of the six Sahelian samples, and similar levels of diversity were estimated, as indicated by gene diversity (h) values ranging from 0.72 to 0.78, and nucleotide diversity (π) values ranging from 0.0018 to 0.0019 (Table 2). No departure from Hardy-Weinberg equilibrium, nor from selective neutrality and demographic equilibrium (with any of the Ewens-Watterson homozygosity or Fu’s *F*_s_ tests) was found (all *P*-values > 5 %; results not shown). On another hand, a significant departure from the neutral equilibrium model in favor of overdominant selection was found with Tajima’s *D* test for the Fulani nomads from the Banfora area (*D* = 2.647, *P* = 0.006, Table 2). This value remained significant after Bonferroni correction for multiple testing. We note that in the five other tested populations, estimates of *D* were all positive although not significant.

### *NAT2* diversity in African populations

The complete list of sixty-one *NAT2* haplotypes detected in the 39 African samples of coding-exon sequences is provided in Additional file 3: Figure S2 and Additional file 4: Table S1. Among these, seventeen haplotypes are newly described (notably due to the inclusion of samples from the 1000 Genomes Project [53]), and were assigned new names by the official *NAT2* nomenclature committee (Additional file 4: Table S1).

Analysis of estimated diversity levels for the 870 bp *NAT2* coding exon evidenced substantial variation between populations, ranging from 0.65 to 0.91 for expected heterozygosity (h), and from 0.0022 to 0.0033 for nucleotide diversity (π) (Additional file 5: Table S2). Heterozygosity levels estimated for the six Sahelian populations are located in the lower half of the distribution (h varying from 0.71 to 0.78). For nucleotide diversity instead, four of our Sahelian samples display intermediate values (around 0.0029), while the Fulani from Bongor display a slightly lower value (0.0028), and the Kanembou a slightly higher one (0.0030). However, these estimates are associated with large standard deviations thus precluding finding significant differences in diversity levels between populations. This is especially the case of many of the published datasets, which include samples of less than 20 individuals. In support of this, we found a high and significant correlation between sample size and number of haplotypes detected (*r* = 0.738, *P* < 0.00001, Additional file 6: Figure S3), and it remained significant even after removal of the four samples with sizes larger than 50 individuals (*r* = 0.398, *P* = 0.018).

Substantial variation in frequency distributions of *NAT2* haplotypes was observed among African populations, particularly so for slow haplotypes NAT2*5B and *6A, and the fast one NAT2*12A (Additional file 7: Figure S4). The variance in haplotype frequency among African populations is highest for NAT2*5B (0.018, Additional file 8: Figure S5). This low-activity haplotype is indeed generally more frequent in populations living in the Sahel or in the dry savannas of East Africa (more than 35 % on average) than in populations living to the south of it (less than 19 % on average), such as the Yoruba or the Pygmy populations (Additional file 7: Figure S4). NAT2*6A, also a slow haplotype, displays a similar trend. Conversely, the fast haplotype NAT2*12A displays a somewhat opposite trend, in that it is frequent in the Pygmy populations and in the San as well (from 22 % to 48 %), whereas its frequency is lower than 14 % in most Sahelian and East African populations. It is also rather frequent in those two Yoruba samples of small size (28 % and 21 % in the YRB and YO, respectively, both samples with less than 20 individuals), but not in the larger sized ones (9 % and 6 % in the YRI and YOR, respectively).

When considering only the 870 bp of the *NAT2* coding exon, a positive and significant Tajima’s *D* value was found again for the Fulani from Banfora, as well as for the Fulani from Ader, the Fulani tested by Mortensen et al. [42], the Mbuti Pygmies, and the Egyptians (Additional file 5: Table S2). These five rejection cases result from an excess of intermediate frequency variants (i.e., positive *D* values ranging from 1.92 to 2.65). For the Mbuti Pygmies, a significant excess of observed heterozygosity was detected also with the Ewens-Watterson test (*P* = 0.0463), thus leading to the rejection of selective neutrality and demographic equilibrium. However, none of these results remained significant after Benjamini and Hochberg false discovery rate (FDR) adjustment for multiple testing.

### *NAT2* population structure in Africa

To investigate for the presence of a genetic structure differentiating populations with distinct food-producing subsistence modes (and for the influence of samples of small size on the results as well), analyses of population structure were performed on three subsets of the population data (Table 1), namely: (1) on the African populations dataset (AFR, i.e., 38 samples, excluding African Americans), (2) on the African food-producing populations dataset (FP, i.e., 29 samples, excluding hunter-gatherers), and (3) on an African food-producing populations dataset comprising only those samples with size ≥ 20 individuals (FPLS, i.e., 13 samples, excluding both hunter-gatherers and all other samples with sizes smaller than 40 chromosomes). Taking into account the molecular diversity of *NAT2* sequences (in terms of the number of pairwise differences between *NAT2* haplotypes), the global level of population structure estimated for the African continent is of 3.3% (AFR dataset, Φ_ST_ = 0.033, *P*< 0.0001), which means that 3.3% of the total genetic variation is attributed to differentiation among populations, while 96.7% is due to differences among individuals within populations. In the AFR dataset, 33.4 % of the pairwise genetic distances between populations were found to be significant at the 5 % level. This proportion drops to 19.5 % in the FP dataset, thus suggesting substantial *NAT2* genetic differentiation both among hunter-gatherer populations and between hunter-gatherer and food-producing societies. Consistently, the proportion of genetic variance explained by differences among populations drops to 2.2% in the FP dataset, but it is still significant (FP dataset, Φ_ST_ = 0.022, *P*< 0.0001). Thus, while differentiation of hunter-gatherer populations contributes to *NAT2* population structure in the African continent, population structure among food-producing communities is also significant. Among the non-significant pairwise genetic distances, a rather large proportion could actually be due to lack of power in significance testing because of small sample sizes. Indeed, the proportion of significant distances increases again to 34.6% in the FPLS dataset, i.e., in the dataset that excludes food-producing population samples of less than 20 individuals. In line with this, the proportion of genetic variance explained by differences among populations also raises to 2.6% (for the FPLS dataset, Φ_ST_ = 0.026, *P* < 0.0001), thereby suggesting that the inclusion of samples of small size in the AFR and FP analyses leads to a lowering of the power to differentiate populations.

Most of the significant pairwise genetic distances in the FPLS dataset differentiate the Yoruba and Akele populations from the others (Additional file 9: Figure S6). Conversely, none of the six Sahelian populations was found significantly differentiated from the others, irrespective of language affiliation or lifestyle, and most of the genetic distances between our samples and the East African samples were also found statistically not significant.

Accordingly, no correlation of genetic distances with geographic distances was found for any of the AFR and FP datasets (*r* = 0.054, *P* = 0.223, and *r* = −0.012, *P* = 0.531, respectively), whereas the Mantel test with the FPLS dataset led to a significant negative correlation (*r* = −0.218, *P* = 0.034). This somewhat surprising result stems from both a set of large genetic distances between populations located in close geographic proximity and a set of small genetic distances between populations geographically far apart (Additional file 10: Figure S7), and does not fit the expectation of an isolation-by-distance model of evolution. In line with this, no significant correlogram of Moran’s *I* spatial autocorrelation indices was found with any of the *NAT2* haplotypes observed in Africa, except for haplotype NAT2*5C, a rather infrequent haplotype.

### Association of *NAT2* genetic structure with geography, culture, or climatic zone and biome

Hierarchical analyses of molecular variance (AMOVA) were carried out to gain further insight into a possible association of the genetic structure of populations with factors related to their demographic and cultural history. Four categorization criteria were tested (Table 1): geography, language, subsistence mode and ecoregion (biome). The results highlight a marked genetic structure associated with the fourth categorization criterion that considers whether populations live within the dry savanna biome or outside of it (Table 3). Indeed, with a classification of populations in ecoregions, Φ_CT_ indices were found high and significant for all three data subsets (Φ_CT_ of 2.3 %, 3.6 % and 5.4 %, for AFR, FP, and FPLS, respectively, all *P*-values < 0.05 and remaining significant after Bonferroni correction for multiple testing) and greater than Φ_SC_ indices in all cases (corresponding Φ_SC_ for AFR, FP and FPLS of 2.2 %, 0.7 % and 0.6 %, respectively, all *P*-values < 0.05). Significant genetic structure was also detected with a classification according to subsistence strategy, but under this categorization criterion and for each of the three datasets, the Φ_CT_ index is lower than the Φ_SC_ index, thereby meaning that more differentiation is found among populations in groups with matching subsistence mode than between those groups, even if only slightly more so. Finally, the results indicate that neither differentiation among geographic groups nor among linguistic groups does associate with the genetic structure of populations displayed by *NAT2* sequences, since Φ_CT_ indices were never found significant for any of the AFR, FP, or FPLS datasets (Table 3 and Additional file 2).

**Table 3:** Analysis of molecular variance (AMOVA) under four criteria of classification of populations, based on the 870 bp long *NAT2* coding exon

				Percentage of variation					
Dataset^1^	Categories grouping^2^	Number of population samples	Number of groups	Between groups	Between populations within groups	Fixation indexes			
						Φ_CT_	*P*-value^3^	Φ_SC_	*P*-value^4^
AFR	Geo^5^	37	3	−0.01^6^	3.23	−0.00012^6^	0.3722	0.03230	**<0.0001** (**0.0004**)
	Lang	38	4	0.43	3.04	0.00425	0.1323	0.03053	**<0.0001** (**0.0004**)
	Subsist	38	4	1.82	2.02	0.01819	**0.0003** (**0.0012**)	0.02055	**<0.0001** (**0.0004**)
	Clim	38	2	2.35	2.11	0.02345	**0.0001** (**0.0004**)	0.02161	**<0.0001** (**0.0004**)
FP	Geo^5^	28	3	−0.04^6^	2.21	−0.00037^6^	0.4572	0.02207	**<0.0001** (**0.0004**)
	Lang	29	3	−0.21^6^	2.30	−0.00207^6^	0.6201	0.02297	**<0.0001** (**0.0004**)
	Subsist	29	3	1.16	1.58	0.01156	**0.0251** (0.1004)	0.01600	**0.0001** (**0.0004**)
	Clim	29	2	3.57	0.71	0.03572	**<0.0001** (**0.0004**)	0.00739	**0.0170** (0.0680)
FPLS	Geo	13	3	−0.53^6^	2.99	−0.00527^6^	0.7238	0.02974	**<0.0001** (**0.0004**)
	Lang^7^	12	2	−0.45^6^	2.84	−0.00447^6^	0.5612	0.02832	**<0.0001** (**0.0004**)
	Subsist	13	2	1.20	1.99	0.01202	0.0530	0.02016	**<0.0001** (**0.0004**)
	Clim	13	2	5.40	0.54	0.05403	**0.0035** (**0.0140**)	0.00568	**0.0462** (0.1848)

^1^ As described in Methods, the three population data subsets are AFR: 38 samples, excluding the Americans of African ancestry (ASW of [53], see Table 1); FP: 29 samples of African food-producing populations; FPLS: 13 samples of African food-producing populations with sample size ≥ 20 individuals. Thus, the ASW sample was not considered in any of the AMOVA analyses

^2^ Categories as in Table 1 : classification according to geographical region (Geo), subsistence mode (Subsist), linguistic affiliation (Lang), and ecoregion (Clim), namely climatic zone and biome, which defines the fourth categorization criterion that considers whether populations live within the dry savanna biome or outside of it

^3^ Significance of the Φ_CT_ index and of the corresponding percentage of variation due to differences between groups. Significant *P*-values (i.e., <5 %) are shown in bold, and adjusted *P*-values after Bonferroni correction for multiple testing (here, four tests) are provided in brackets

^4^ Significance of the Φ_SC_ index and of the corresponding percentage of variation due to differences between populations within groups. Significant *P*-values (i.e., <5%) are shown in bold, and adjusted *P*-values after Bonferroni correction for multiple testing (here, four tests) are provided in brackets

^5^ Only three geographical regions are considered here (Western, Central and Eastern, Table 1) because the fourth region (Northern) is represented by one population sample only (EGY)

^6^ Because variance components in AMOVA are actually defined as covariances, negative values can occur [95]. A negative Φ_CT_ value would be expected if gene copies were more correlated between groups than between populations within groups. However, none of the negative Φ_CT_ values in the table are statistically significant, thus indicating that they are equal to zero

^7^ Only two linguistic families are considered here (Niger-Congo and Nilo-Saharan, Table 1) because the third family (Afro-Asiatic) is represented by one population sample only (SOM)

**Fig. 2:**
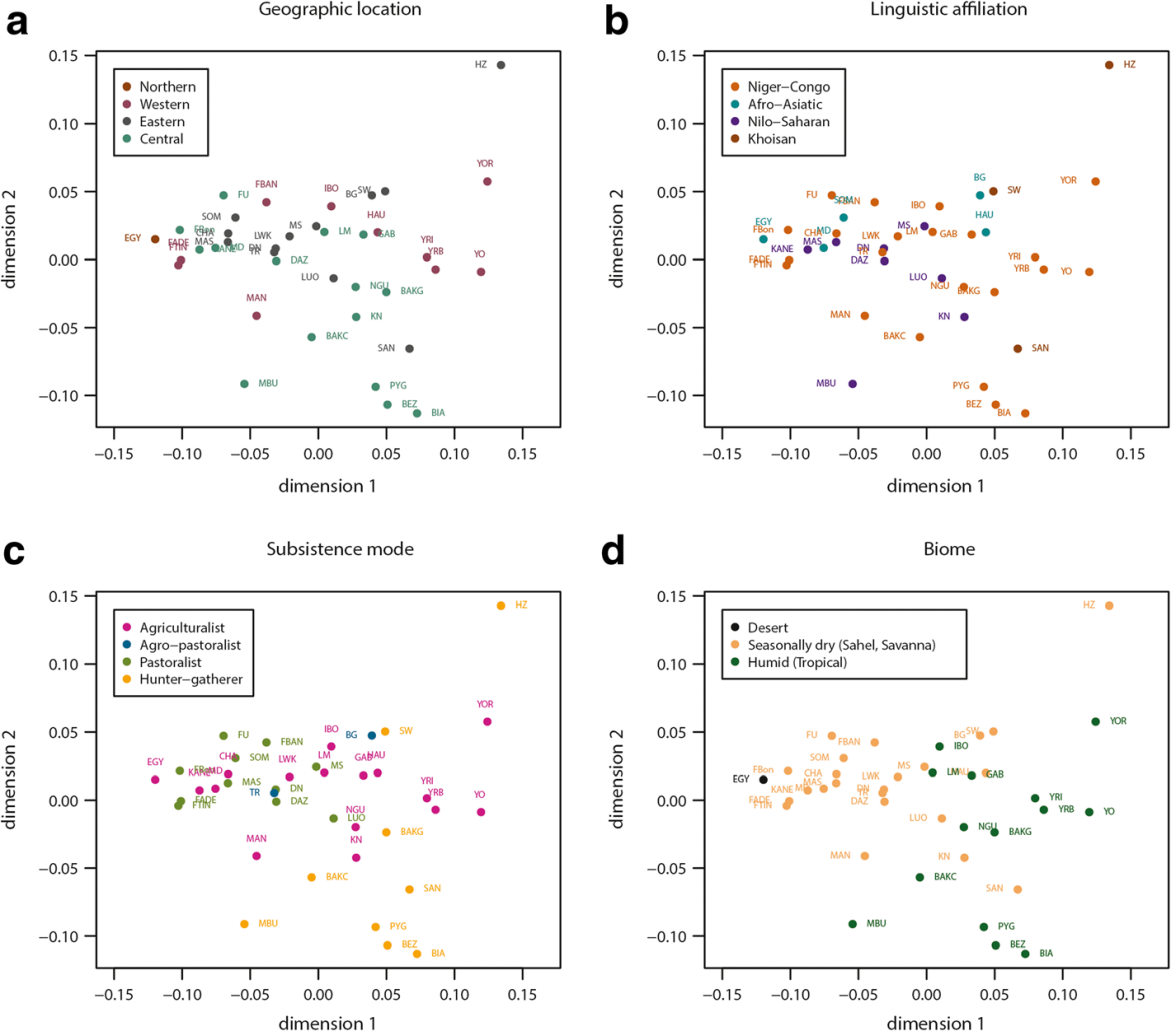
MDS plot of pairwise Reynolds genetic distances between the 38 populations of the AFR dataset. The Stress value is 0.071. The same plot is reproduced 4 times, with populations color-coded according to: (**a**) geographical region, (**b**) linguistic affiliation, (**c**) subsistence mode, and (**d**) biome (see text)

The two-dimensional plots resulting from the nonmetric multidimensional scaling (MDS) analyses of genetic distances among populations are shown in Fig. 2 for the AFR dataset, and in Additional file 11: Figure S8 and Additional file 12: Figure S9 for the FP and FPLS datasets, respectively. In each of these figures, the MDS plot is displayed four times, with populations being highlighted according to our four categorization criteria, namely geography, language, subsistence mode and biome. Consistent with the AMOVA results, categorization according to the environment seems to better fit with the location of populations in the plots than any of the three other criteria (see also Additional file 2).

### NAT2 Phenotypes

**Fig. 3:**
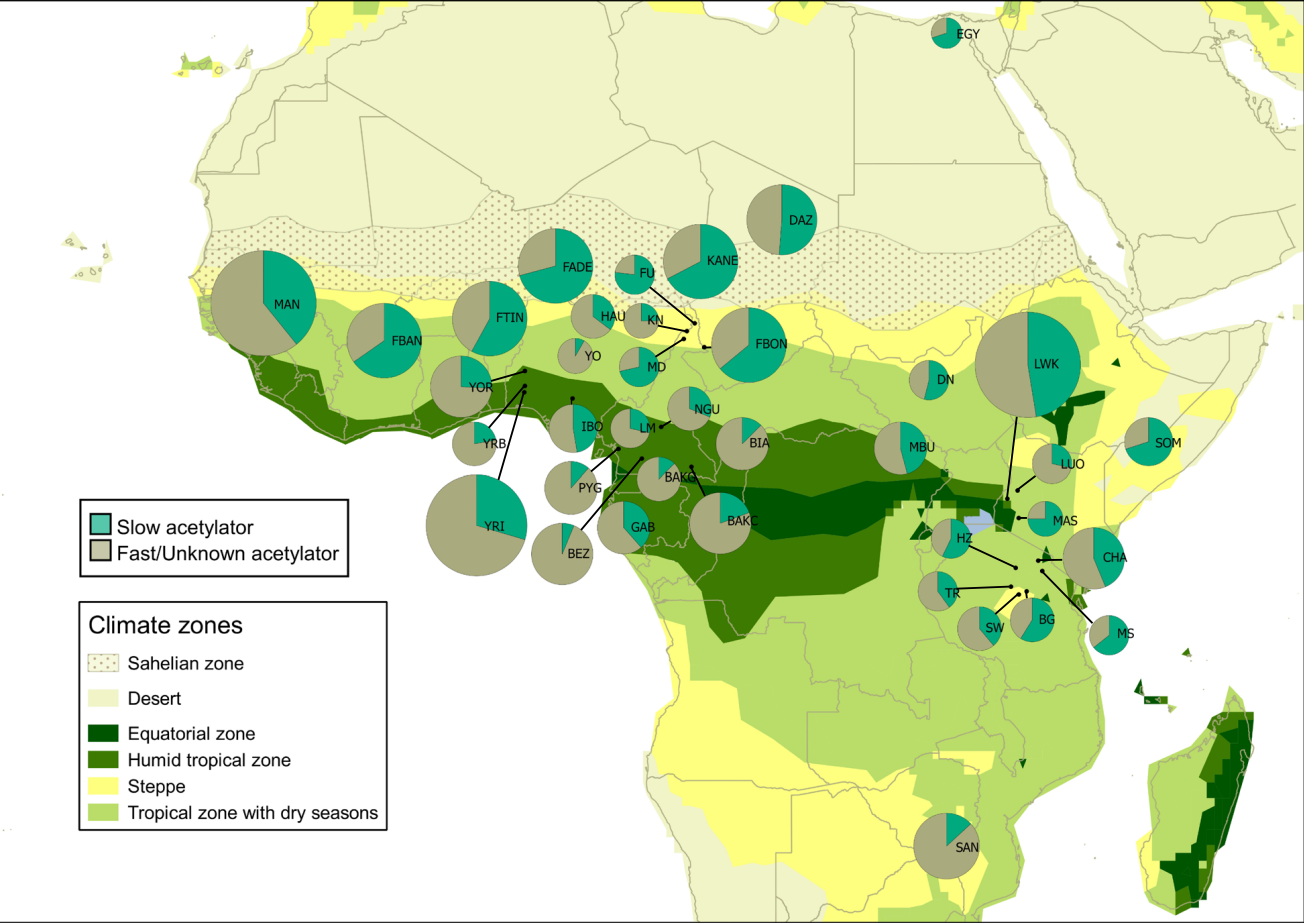
Map showing the frequency distributions of predicted *NAT2* phenotypes in African populations screened for sequence variation in the coding-exon (pie charts are proportional to sample size). Map created with the QGis open source software [98], with climatic zones defined according to [99]

**Fig. 4:**
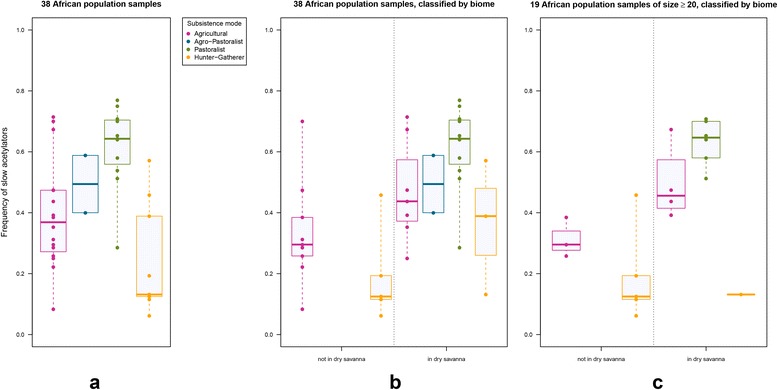
Boxplots of predicted prevalence of slow acetylators in populations classified according to subsistence mode and further to ecoregion. Each box extends from the first to the third quartile and displays the median (thick line), and the dispersion of populations’ individual values (filled circles) is shown by dashed lines. (**a**) Thirty-eight African populations (AFR dataset); (**b**) same dataset crossed with biome information, i.e., separating populations living in the seasonally dry zones (Sahel, Savanna) from those living in the humid tropical and equatorial zones; (**c**) same cross-analysis as in (**b**) but excluding population samples of less than 20 individuals

The frequency of slow acetylators in each population sample was predicted from individuals’ *NAT2* genotypes (i.e., diplotypes). Predicted proportions of slow acetylators in African populations (Additional file 13: Table S3) are displayed on a map in Fig. 3. Boxplots of predicted prevalence of slow acetylators (Fig. 4) indicate higher median proportion of slow acetylators for pastoralists than for the other subsistence modes (pastoralists > agro-pastoralists > agriculturalists > hunter-gatherers, Fig. 4a), although the variances of these proportions among populations are very large. On another hand, slow acetylation is more frequent in populations living/dwelling in the Sahelian belt or in the dry savanna surrounding it (including in the East African dry savanna zones) than in populations living to the South of it (Fig. 3), apparently irrespective of their subsistence strategy (Fig. 4b). Actually, crossing the information on subsistence mode with that on biome suggests higher slow acetylation in agricultural and hunter-gatherer populations living in the seasonally dry zones (Sahel, Savanna) than in those living in the humid tropical and equatorial zones. This trend seems even more marked for agriculturalists when only population samples of at least 20 individuals are considered (Fig. 4c).

Kruskal-Wallis tests for homogeneity of slow acetylation frequency among groups corroborated these observations (Table 4). Neither groups defined by geographic locations (Geo) nor groups defined by linguistic families (Lang) associate with differences in slow acetylation prevalence among populations. In turn, a significant difference in the frequency of slow acetylators was found among populations relying on different subsistence strategies (Subsist, *P* = 0.0014). This difference remains both when hunter-gatherers and when small samples are excluded from the analysis (FP and FPLS datasets, *P* = 0.0161, and *P* = 0.0152, respectively). When hunter-gatherers are excluded (FP dataset), only three subsistence modes are represented in the data, namely agriculturalists, agro-pastoralists, and pastoralists (Table 1). When small samples are excluded (FPLS dataset), only agriculturalists and pastoralists are represented, thus implying that slow acetylation prevalence is significantly higher among pastoralist than among agriculturalist populations. These results were confirmed by pairwise Wilcoxon tests of equality in average slow acetylation frequencies among subsistence strategies (Additional file 14: Table S4). A significant difference in slow acetylation prevalence was consistently found between pastoralists and agriculturalists (and between pastoralists and hunter-gatherers as well). Nevertheless, high prevalence of slow acetylation was also predicted for several agriculturalist populations from dry savanna regions, such as the Kanembou (67.3 %, 95 % confidence interval = [55.1%; 79.6 %]) and Luhya (47.4 %, 95 % confidence interval = [38.1 %; 56.7 %]), thus suggesting a possible association of acetylation status with ecoregion, namely with the climatic zone and biome populations live in (Fig. 4c). In line with this observation, a highly significant difference in average slow acetylation frequency was consistently found between populations living within the dry savanna biome or outside of it (Clim, *P* = 0.0003, *P* = 0.0054, and *P* = 0.0112, for the AFR, FP, and FPLS datasets, respectively, Table 4), irrespective of subsistence strategy.

**Table 4:** Kruskal-Wallis test for equality of frequency of the slow acetylation phenotype across geographical regions, linguistic families, subsistence modes, and climatic zones

Dataset^1^	Categories grouping^2^	Number of populations	Number of groups	Kruskal-Wallis H statistic	*P*-value^3^
AFR	Geo^4^	37	3	2.38	0.3039
	Lang	38	4	7.13	0.0680
	Subsist	38	4	15.62	**0.0014** (**0.0056**)
	Clim	38	2	13.17	**0.0003** (**0.0012**)
FP^5^	Geo^4^	28	3	2.52	0.2843
	Lang	29	3	3.61	0.1649
	Subsist	29	3	8.25	**0.0161** (0.0644)
	Clim	29	2	7.74	**0.0054** (**0.0216**)
FPLS	Geo	13	3	0.33	0.8480
	Lang^5^	12	2	1.15	0.2827
	Subsist	13	2	5.90	**0.0152** (0.0608)
	Clim	13	2	6.43	**0.0112** (**0.0448**)

^1^ As described in Methods, the three population data subsets are AFR: 38 samples, excluding the 1KG Americans of African ancestry (ASW, see Table 1); FP: 29 samples of African food-producing populations; FPLS: 13 samples of African food-producing populations with sample size ≥ 20 individuals. Thus, the ASW sample was not considered in any of the Kruskal-Wallis tests

^2^ Categories as in Table 1 : classification according to geographical region (Geo), subsistence mode (Subsist), linguistic affiliation (Lang), and ecoregion (Clim), namely climatic zone and biome, which defines the fourth categorization criterion that considers whether populations live within the dry savanna biome or outside of it

^3^ Significant *P*-values (i.e., <5 %) are shown in bold, and adjusted *P*-values after Bonferroni correction for multiple testing (here, four tests) are provided in brackets

^4^ Only three geographical regions are considered here (Western, Central and Eastern, Table 1) because the fourth region (Northern) is represented by one population sample only (EGY)

^5^ Only two linguistic families are considered here (Niger-Congo and Nilo-Saharan, Table 1) because the third family (Afro-Asiatic) is represented by one population sample only (SOM)

Single factor analyses of variance (ANOVA) corroborated the results of the Kruskal-Wallis rank sum tests in that predicted slow acetylation frequency is observed to differ significantly between populations classified both by ecoregion (Clim) and subsistence mode (Subsist), but not by geography (Geo) or linguistic classification (Lang) (Additional file 15: Table S5). Moreover, ecoregion and subsistence mode were shown to be independently associated with differences in mean predicted slow acetylation frequency by multi-factor ANOVAs, as no significant interaction between these two factors (categorization groups) was found. Finally, Tukey’s honest significant difference (HSD) tests for multiple comparisons of means confirmed the significant difference in predicted frequency of slow acetylators between populations living/dwelling in seasonally dry zones (Sahel, savanna biome) and those living in humid tropical and equatorial zones (i.e., “Living outside”), as well as between populations relying on different subsistence modes, notably between those relying on pastoralism and those relying on agriculture (Additional file 15: Table S5).

Taken together, our results thus indicate that factors associated both to the ecoregion in which populations have been living/dwelling and the mode of subsistence on which they have been relying independently explain a significant proportion of NAT2 phenotypic diversity in Africa.

## Discussion

In this study, we have generated 1,396 bp of sequence encompassing the *NAT2* coding exon in 574 chromosomes sampled in six Sahelian populations. The activity of the enzyme encoded by this highly polymorphic gene is of clinical importance as it influences individuals’ physiological response to the absorption of numerous chemical compounds present in the diet and the environment, including several nowadays useful medications, and it is also involved in cancer development risk [37]. Five of the populations studied rely on pastoralism as a mode of subsistence, four of which are the Fulani nomads from Niger, Burkina Faso and Chad, all speaking dialects of a Niger-Congo language (Fulfulde) [54, 55], whilst in the fifth one, the Daza from Chad, a Nilo-Saharan language (Dazaga) is spoken [54, 56]. The sixth population sample studied represents the Kanembou, a sedentary population, also from Chad, but relying on agriculture, and speaking a Nilo-Saharan language [54, 57].

### Intermediate to low *NAT2* molecular diversity in Sahelian populations

Twenty-one haplotypes were parsimoniously inferred for the six Sahelian samples, of which three haplotypes not yet described to the best of our knowledge (NAT2*12N, NAT2*13D and NAT2*14K, Fig. 1). In order to compare our newly generated dataset with published *NAT2* sequences from other African samples, we restricted the analysis to the 870 bp of the *NAT2* single coding exon. We have thus shown that levels of genetic diversity in the six Sahelian populations are amidst the low to intermediate range of values in the scale of *NAT2* polymorphism known for the African continent, especially so when measured by expected heterozygosity (Additional file 5: Table S2). This result could be due to faster genetic drift in these populations compared to others, which is congruent with the idea, based on analyses of polymorphisms in uniparentally transmitted DNA (mitochondrial DNA and Y chromosome) showing that some Sahelian populations experienced a demographic contraction in their history [54, 55]. Actually, in line with mitochondrial DNA (mtDNA), *NAT2* diversity was found somewhat lower in the Fulani pastoral groups than in the Kanembou agriculturalists (both when measured by expected heterozygosity and by nucleotide diversity), consistent with recent genomic results that inferred smaller expansion rates for pastoral populations than for those having adopted agriculture [58, 59]. Moreover, while nearly none of the Ewens-Watterson homozygosity or Fu’s *F*_S_ tests on *NAT2* variation rejected the null hypothesis of selective neutrality and demographic equilibrium, Tajima’s *D* tests were found significantly positive for three Fulani groups, although not anymore after correction for multiple testing. Nevertheless, for one of those Fulani groups (FBAN), Tajima’s *D* was positive when tested on the entire sequenced segment of 1,396 bp, and it remained significant after correction (Table 2). Thus, this trend towards Tajima’s *D* positive values could also point to a reduction in population size [60], particularly so in the pastoral nomads. However, irrespective of subsistence mode, most African populations analyzed here displayed the same trend towards positive *D* values, which are also expected if the gene is submitted to balancing selection. The signature left by balancing selection should be detected by the Ewens-Watterson homozygosity test, since this selective regime should lead to an excess of heterozygotes, as has been shown for the Human Leukocyte Antigen (HLA) loci [61]. As already stated, among the 39 Ewens-Watterson tests performed on the complete population dataset, a single case of significant rejection of neutrality was found for the Mbuti Pygmies (Additional file 5: Table S2), because of heterozygosity in excess (which would fit the balancing selection model), but not anymore after adjustment for type I error rate. While all other tests were not significant, the proportion of cases in which the observed heterozygosity was lower than the expected was of nearly 40% (i.e., 15 out of 38). However, positive *D* values can also result from the presence, in the populations’ gene pools, of several molecularly rather distant haplotypes at frequencies higher than expected under neutrality. Such frequency distributions can develop if several standing variants become submitted to positive directional selection [62]. This interpretation was favored in a recent study that screened variation of the entire *NAT2* locus sequence (ca. 10 kb) in 14 world populations from the 1000 Genomes Project [63], but no pastoral population was represented in that collection.

### The genetic structure of *NAT2* in Africa is neither associated to geography nor to linguistics

Consistent with previous studies [24], we found a significant genetic structure of *NAT2* in Africa (Φ_ST_ = 3.3 % for the AFR dataset of 38 populations). Here we further showed that this structure is not only attributable to the genetic differentiation of hunter-gatherer populations but is also found among African food-producing communities, as attested by the significant Φ_ST_ value of 2.6 % for the FPLS dataset of 13 populations. This significant genetic structure is mainly due to the variation in frequencies of three major haplotypes, two of which are categorized as decreased function alleles (NAT2*5B and NAT2*6A), which are particularly frequent in populations living in the dry savanna biome, and one fast allele (NAT2*12A), more frequently detected outside this region (i.e., in populations living in tropical humid environments) than within it. Accordingly, both the AMOVA (Table 3) and MDS analyses (Fig. 2 and Additional file 11: Figure S8 and Additional file 12: Figure S9) indicate that neither differentiation among geographic groups nor among linguistic groups does associate with the genetic structure of populations displayed by *NAT2* sequences, contrarily to what is known for polymorphisms at other loci of the genome, such as mtDNA [64], Y chromosome and classical polymorphisms [55, 65, 66], or even for genome-wide variation [67, 68]. Furthermore, the genetic structure of *NAT2* in Africa does not strongly associate with subsistence strategy either, mainly because most Sahelian and East African populations display little to no genetic differentiation between them (Additional file 9: Figure S6), although both regions are populated by nomadic or semi-nomadic pastoralist and sedentary agriculturalist communities. Actually, this low genetic differentiation between most Sahelian and East African populations suggests a possible climate and biome link that is supported by the significant association of *NAT2* genetic structure with a classification of populations according to ecoregions. However, in contrast to biological functions for which the link with environmental pressures is obvious, such as immunity and pathogens [69–72], a climate and biome related factor that would affect *NAT2* evolution, if any, remains to be determined [25].

### Differences in slow acetylation prevalence across the southern Sahelian limit

The frequency of slow acetylators in each population sample was predicted from individuals’ *NAT2* genotypes (i.e., diplotypes). Kruskal-Wallis tests of homogeneity in proportions of slow acetylators among groups of populations did not evidence significant variation between geographic groups, nor between linguistic groups. In turn, significant difference of slow acetylators were found between subsistence strategies, and specifically between pastoralist and agriculturalist populations (Table 4, *P* = 0.016 and *P* = 0.015, for the FP and FPLS datasets, respectively; see also Additional file 14: Table S4 and Additional file 15: Table S5). Considering only those populations for which sample sizes included at least 20 individuals, average frequency of slow acetylators among pastoralists is of 63.2 % (±7.5), while it is of 41.6 % (±13.6) among agriculturalists (Additional file 13: Table S3 and Fig. 4). The higher standard deviation estimated for the latter group reflects a high variance in slow acetylator frequencies among agriculturalist populations such as the Yoruba (less than 30 %) and the Kanembou (more than 67 %). By contrast, among pastoralists, these frequencies vary from more than 50 % in the Daza to around 70 % in the Somali and the Fulani from Ader. For comparison, slow acetylators average to 18.1 % (±14.2) among hunter-gatherers.

Similarly to the finding of a significant difference in proportions of acetylation phenotypes between sedentary farmers and nomadic pastoralists in Central Asia [43], these results are compatible with the hypothesis of a differential evolution of *NAT2* acetylation capacity according to lifestyle among food-producing societies in Africa. It is thus tempting to suppose that the driving selective force behind this differential evolution is to be found in the development of dietary differences due to lifestyle. Polymorphisms in the *NAT2* gene or downstream from it and tagging its phenotypic expression have been recently identified by genome-wide association studies for their role in the metabolism of sugars and carbohydrates [73, 74], or in that of lipids [75, 76], thus making a strong case for dietary influences. However, we have also found a significant difference in frequencies of the slow acetylator phenotype across the southern Sahelian limit, thus showing that such difference is actually enclosed in the zones defined by climate and biome in which the populations are living. Irrespective of lifestyle (whether pastoralist, agriculturalist, or even agro-pastoralist), we found significantly higher proportions of slow acetylators in populations living in the Sahelian and tropical with dry seasons zones than in those living in the humid tropical and equatorial zones (Table 4, *P* = 0.005 and *P* = 0.011, for the FP and FPLS datasets, respectively). Multi-factor ANOVAs further showed that no significant interaction between subsistence mode and ecoregion explained the observed differences in predicted mean slow acetylation frequency (Additional file 15: Table S5). Altogether, these results point to a possible independent influence of dietary habits on one hand (as reflected by subsistence modes), and of the chemical environment on the other hand (as reflected by climatic zones), in the evolution of *NAT2* diversity. Thus, this hypothesis does not necessarily call for the existence of specific dietary components associated with subsistence modes, and hence has the advantage of being compatible with the opposite pattern of variation observed in sub-Saharan Africa with respect to Central Asia, where higher slow acetylation prevalence is observed in sedentary agriculturalists than in nomadic pastoralists [43]. Further population sampling will thus be needed to substantiate it. At present, we can only acknowledge that the difference in slow acetylation frequency between African sedentary agriculturalist populations living within the dry savanna biome or outside of it was not found significant (Wilcoxon rank sum test *P*-values of 0.11 and 0.057, respectively, for the comparison of the sixteen agriculturalist samples, and that of the seven agriculturalist samples of size ≥ 20 individuals).

### Causative factors driving *NAT2* evolution

Consistent with previous investigations on *NAT2* molecular variation [42] (and references therein), no statistically significant signal of selection was found in our datasets after correction type I error rate. This suggests that if positive directional selection for slow-causing *NAT2* haplotypes in specific dietary and environmental conditions has been acting on the genetic evolution of populations, such selective pressures might have acted on multiple standing genetic variation rather than on specific new *NAT2* mutations [25, 26, 38, 43, 77]. We acknowledge indeed that classical selective neutrality tests on population molecular data (such as the Ewens-Watterson homozygosity, Tajima’s *D* and Fu’s *F*_S_ tests used here) are known to have little power to detect a signal of selection on standing genetic variation [62, 78]. Thus, the possibility exists that the genetic structure of African food-producing populations inferred through the analysis of *NAT2* molecular diversity, which differentiates populations from distinct climatic zones, could result from differentiated selective pressures on standing variation related to the xenobiotic environment of distinct climate and biome zones, and explain the close genetic proximity of Sahelian populations with East African populations independently of their lifestyle.

Alternatively, archaeological evidence suggests that the Sahel could have been first inhabited only by pastoralists, and adoption of farming by some populations would represent a recent, secondary change [47, 48]. Thus the lack (or nearly so) of significant genetic structure of *NAT2* molecular variation among Sahelian populations would be compatible with the hypothesis of differentiated selective pressures on standing variation related to xenobiotic intake of distinct dietary habits. This hypothesis has the advantage of explaining the significant difference in frequencies of the slow acetylator phenotype observed between African pastoralists and agriculturalists. Consequently, either secondary adoption of farming by formerly pastoralist populations should also be postulated for some East African populations, such as the Luhya from Kenya (of the 1000 Genomes Project [53]), or a rather common occurrence of gene flow between pastoralists and agriculturalists populations in East Africa should be envisioned. This latter case is compatible with the complex history of East African populations evidenced for mitochondrial DNA [64], as well as at the genome-wide level [68, 79–81].

Finally, as discussed in [24], it is also possible to envision that no selective constraint has acted on the evolution of *NAT2*, notably if the function of the enzyme encoded by this gene is redundant with other enzymes such as NAT1. In this case, the low genetic differentiation observed between Sahelian and East African populations could be due to a recent common origin. Under this last hypothesis, we would expect a consistent pattern of variation of *NAT2* with the genetic structure inferred from other independent genetic markers in the genome, thus pointing to the demographic history of these populations rather than to specific selective pressures having acted on *NAT2* evolution. This last alternative is not favored at present, given the patterns of variation known for other genomic segments, as discussed above, but it calls for further investigation because it would allow disentangling the effects of demographic and selective processes in the evolution of *NAT2*.

Our analysis suffers of course of some drawbacks. Firstly, as already stated, because it makes the simplistic assumption of a discrete categorization model of acetylation phenotypes (i.e., slow versus intermediate/fast acetylators) on the basis of genotypes, although heterogeneity in these categories has been recently demonstrated [33, 34]. For instance, acetylation status was found to vary significantly between the three genotypes formed by the two slow alleles NAT2*5 and NAT2*6, with NAT2*5 homozygotes being faster acetylators than NAT2*5/NAT2*6 heterozygotes, and the latter displaying faster acetylation than NAT2*6 homozygotes. In addition, the existence of variation in acetylation capacity due to polymorphisms outside the coding-exon of the *NAT2* gene is not considered here, although we know that regulatory polymorphisms could alter levels of protein expression and thereby acetylation activity. Besides, the existence of epistatic relationships between the expression of *NAT2* and its ortholog *NAT1* has been described [82], thus further emphasizing the complexity of the acetylation function in the organism and its evolution.

Secondly, the high level of polymorphism displayed by *NAT2* in human populations is probably very often underestimated with the samples sizes currently in use, as shown by the high and significant correlation of the latter with the number of alleles detected, at least for African populations (Additional file 6: Figure S3). Interestingly, we found that this correlation holds both for slow-causing variants (*r* = 0.655, *P* < 0.00001) and variants with unknown effect on phenotype as well (*r* = 0.605, *P* < 0.00001), but not for fast acetylation alleles (Additional file 16: Figure S10). This last result suggests that many more slow-causing variants could exist, probably at low frequencies, which is compatible with the hypothesis of a relaxation of functional constraints on *NAT2* in the course of human evolution, notably in those populations that adopted a food-producing mode of subsistence, where these variants are more frequent.

Thirdly, we should also consider that the discrete lifestyle categories in which we have classified our population samples (pastoralists, farmers or agro-pastoralists) are rather gross approximations of much more complex subsistence strategies. Extant cultures are not mono-subsistent as their diets also rely on various complementary modes of food supply, such as fishing and gathering, and complex networks of food exchange are concurrently used [83, 84]. For instance, some groups among the Kanembou agriculturalists from Chad are specialized in hunting and gathering [85]. Notably, the collection of a wild grass species known as *kreb* and of Spirulina algae known as *dihe’* makes a substantial contribution to the diets of these peoples [86]. At present unfortunately, to the best of our knowledge no accurate estimation of the relative dependence on various forms of subsistence is available for our samples.

Finally, the ecological zones considered in our analyses have been shifting in the past, so that populations living in one zone today might have coped with a different environment several generations ago. Extreme environmental changes are documented specifically in the Lake Chad basin; the current extension of the lake is ~20,000 km^2^ but it is estimated that it was ~350,000 km^2^ some ten thousand years ago [87]. In fact, with the onset of Holocene, the southern Sahara changed into a landscape rich in water resources, as evidenced by archaeological findings of items related to an aquatic life in places where we can barely imagine them today: in addition to direct evidence, such as hippopotamus and crocodile bones in dry river systems, there are findings of harpoons, which people used to hunt these animals [88].

## Conclusion

The pattern of variation of *NAT2* in sub-Saharan African food-producing populations differs from that expected under a model of isolation-by-distance or from those observed with other genetic systems, but it is compatible with the hypothesis that it was shaped by selective pressures linked to the chemical environment in which populations evolved. We have shown that it is possible that differences in xenobiotic environments associate with climates and biomes that oppose arid, seasonally dry regions, such as the Sahel to tropical humid regions, such as around the Gulf of Guinea, hence explaining *NAT2* genetic and phenotypic differentiation across the southern Sahelian limit. However, the possibility exists that differences in xenobiotic environments interacting with NAT2 could also result from differential dietary habits linked to subsistence modes. Under this hypothesis, the genetic similarity between eastern and central-western African populations from the Sahel would then be explained by two non-mutually exclusive processes, namely significant gene flow across the Sahel, or secondary shift from pastoralism to agriculturalism in those Sahelian and East African populations that practice agriculture nowadays. Future studies including measurements of phenotypes and more sampling of populations (with large sample sizes) will certainly shed a new light on these conjectures.

## Methods

### Samples

Biological samples (buccal swabs or saliva samples) were collected during several missions in 2003, 2004, 2005 and 2010 in Niger, Burkina Faso and Chad, from unrelated anonymous volunteers, including only individuals with four grand-parents born to the population. Variability of DNA extracted from these samples was analysed in numerous former studies, such as in [54–57, 89–91].

Fulani nomads, representing the nomadic pastoral lifestyle, were sampled at different places within their geographic range across the Sahelian zone [54, 89]. The westernmost part of the Sahelian belt is represented by Fulani from the western and eastern parts of Burkina Faso, respectively (Banfora area, *n*=49, and Tindangou area, *n*=50). More eastern Fulani groups were sampled in southern Niger (Ader area, *n*=48) and western Chad (Bongor area, *n*=50). As Fulani groups move frequently, the samples were secured in the temporary camps during the dry seasons, when the nomads rested at the southern extremity of their migration routes or transhumance corridors. The semi-nomadic Daza people (*n*=41) were collected in the northernmost regions of Lake Chad basin [56]. The Daza also rely mainly on animal husbandry, although some groups are semi-sedentary, cultivating palms in small oases. The sedentary Kanembou (*n*=49) from the northern fringes of Lake Chad represent agriculturalists in our dataset [54]. Informed verbal consent was obtained from all donors together with sampling authorization from the appropriate state institutions of the respective countries involved, namely from the Ministère des Enseignements Secondaire et Supérieur de la Recherche et de la Technologie (ref. 1534), and the Ministère de la Santé Publique et de la Lutte contre les Endémies, both in Niamey, Niger (ref. 13/2004/CCNE) for sampling in Niger, from the Ministère des Enseignements Secondaire et Supérieur de la Recherche Scientifique, in Ouagadougou, Burkina Faso (ref. 1029) for sampling in Burkina Faso, and from the Centre National d’Appui à la Recherche, in N’Djamena, Chad (ref. 01/CNAR/2010) for sampling in Chad. Ethics approval was required in Niger, and obtained on September 23, 2004, from the Comité Consultatif National d’Ethique (Ministère de la Santé Publique et de la Lutte contre les Endémies, Niamey, Niger). In Burkina Faso and Chad, no ethics approval was required by National Authorities, possibly because ethics commissions were not operating, but the same study protocol was followed in all three countries. Only the geographic location of sampling sites was recorded (i.e., no name or or any other identifiable information about volunteers was asked). An interpreter assisted the sampling collection, since the majority of participants expressed themselves in local languages/dialects, and most of them could not read or write in official language(s). Each participant provided informed consent orally and we repeatedly ensured, by means of the interpreter, that all participants fully understood the purpose of providing their saliva or buccal swabs: investigate the genetic variability of their population and its evolutionary causes. Participants were unrelated healthy adults, age range was 18–55 years, and both males and females were included. As many participants as technical limitations permitted (i.e., materials and costs) were included for sampling.

### DNA samples preparation and *NAT2* sequencing

DNA was extracted from buccal swabs using the method described in [92]. Daza saliva samples were collected with Oragene™ DNA (OG-500) collection kit and extractions were performed according to the manufacturer’s protocol. A segment of ca. 1.5 kb in the region encompassing the 870 bp single coding exon of the *NAT2* gene was amplified and subsequently sequenced in both forward and reverse directions. Two partially overlapping pairs of primers were used (number-named according to the *NAT2* exon): −128F/+914R [24] and +702F/+1373R [42]. Sequencing service was provided by Macrogen, Seoul, Korea. To confirm the detection of mutation 121A>T in a single Fulani individual in heterozygous state, another pair of primers was used (i.e., -15F and +779R of [42]).

### Inference of *NAT2* haplotypes

The sequence haplotypes and their associated maximum likelihood (ML) frequencies were inferred separately for each population sample, using the Bayesian approach based on an approximate coalescent model implemented in the software PHASE v.2.1 [93, 94], and the maximum likelihood (ML) approach based on the expectation-maximisation (EM) algorithm implemented in Arlequin ver. 3.5 [95]. To this end, PHASE and Arlequin were run independently, and the resulting inferences were compared. Because we observed that PHASE results were less stable among runs (slightly different haplotype calls output with different seed numbers but with the same settings) than Arlequin results (identical haplotype calls with the same settings), we chose the latter software to obtain the haplotype calls and their associated ML frequencies (see also Additional file 2).

Following the standards of the official nomenclature of human *NAT2* alleles [37], haplotype NAT2*4 (GenBank accession X14672) was used as a reference for the coding exon. However, because of known variation in the flanking region of the coding exon of NAT2*4 alleles [26, 42, 44], we chose the human genome reference sequence (GRCh37/hg19, http://genome.ucsc.edu/) as a reference for the upstream and downstream flanking regions, and created a NAT2*4/hg19 construct. The sequences generated in this study were thus aligned with this construct, using ClustalW [96]. Two in-house programs were designed so as to (1) identify and map variant positions with respect to the NAT2*4/hg19 construct, and (2) classify inferred phased haplotypes according to the official *NAT2* gene nomenclature.

### Collection of published *NAT2* sequences from African populations

Our new dataset was completed with published *NAT2* sequences from sampled African populations obtained through a comprehensive search of publications. Only populations represented by samples including at least 10 individuals (i.e., 20 chromosomes) were considered. Sequence genotypes from [97], obtained through personal communication with the authors, had to be phased in order to infer sequence haplotypes. The new dataset also comprises *NAT2* sequences reconstructed from the phased variant call file extracted from the 1000 Genomes Project (phase 1, release version 3 of April 2012, [53]), which were obtained with a Python program developed for this purpose. Together with the new 574 *NAT2* sequences generated in this study (i.e., 287 individuals), the complete dataset assembled includes 38 African population samples and one sample of African Americans, totalizing 1,192 individuals (Table 1 and Additional file 1: Figure S1). Geographic maps reporting the samples (Fig. 3, and Additional file 1: Figure S1 and Additional file 7: Figure S4) were created with the QGis open source software [98], and climatic zones were defined according to [99].

### Estimates of diversity within populations

The ML frequency distributions of *NAT2* haplotypes in the six Sahelian population samples and published frequency distributions from other African samples were used to estimate molecular diversity indices (gene and nucleotide diversity), and to test for possible deviation from Hardy Weinberg equilibrium and selective neutrality (the latter through the use of three tests, Ewens-Watterson homozygosity test, Tajima’s *D* test and Fu’s *F*_*s*_ test) with the Arlequin software.

For all tests that revealed at least one significant departure from the null hypothesis in one population, we used R [100] to apply a correction method to control for type I error rate (either Bonferroni, or the less conservative Benjamini and Hochberg FDR when the number of tests exceeded ten), so as to obtain adjusted *P*-values. The R environment was also used to calculate Pearson's product–moment correlation coefficient between samples sizes and observed levels of diversity (e.g., number of haplotypes).

### Estimates of differentiation between populations

The Arlequin software was also used to estimate population pairwise Φ_ST_ values (using the observed number of pairwise differences between haplotypes as a measure of molecular distance, with a 4:1 transition:transversion ratio), test their statistical significance (10,000 permutations) and compute Reynold’s genetic distances, as well as to perform AMOVA analyses to test the significance of population groups’ structures (here with100,000 permutations) according to geographic location, linguistic affiliation, subsistence mode or climatic zone and biome (i.e., ecoregion, following [99]). Under this last classification criterion, all populations living in the Sahel or in areas defined by a tropical with dry seasons climate were grouped into the “dry savanna” biome, whereas those living to the south and west of it, namely in the tropical humid and equatorial zones, were grouped into the “humid” or “desert” biome (Table 1). Matrices of pairwise Reynold’s genetic distances were submitted to nonmetric multidimensional scaling analyses, using the function metaMDS of the vegan package in R. The mantel function of vegan was used to test the significance of correlation coefficients between matrices of pairwise geographic and genetic distances with Mantel tests. Spatial autocorrelation analyses were performed with the PASSaGE software [101].

### Prediction of acetylation capacity and phenotype diversity among populations

Following previous reports [38, 63] (and references therein), the predicted acetylation phenotype associated with a given diploid haplotype combination was categorized as “slow” only if the latter is made of two haplotypes with reported low-activity in the official *NAT2* gene nomenclature. All other combinations (including those made of a low-activity haplotype and an unknown-activity haplotype or a newly described haplotype) were parsimoniously pooled together in a “fast/unknown” phenotype category, thus ensuring conservative estimation of slow acetylation prevalence in each population sample. Ninety-five percent non-parametric confidence intervals of slow-acetylation frequencies were generated by bootstrapping over individuals in each sample with the boot package of R (using 10,000 bootstrap samples). Homogeneity of inferred phenotype frequencies (i.e., “slow” versus “fast/unknown” acetylators) among geographic regions, linguistic groups, subsistence modes, and climatic zones was tested with R by performing Kruskal-Wallis rank sum tests and single/multiple factors ANOVAs, and differences in average slow acetylation frequencies among subsistence modes or between ecoregions were tested with pairwise Wilcoxon rank sum and Tukey’s HSD tests.

## Availability of supporting data

The data sets supporting the results of this article are available within the article and its additional files. The 574 *NAT2 *sequences generated in this study are available under accessions KR231046 to KR231619 in the GenBank database at http://www.ncbi.nlm.nih.gov/genbank/.

## Additional files

Additional file 1: Figure S1. Map showing the location of African populations screened for sequence variation in *NAT2*, including the six Sahelian populations of this study. The ASW sample of African Americans from the 1000 Genomes Project is not located on this map. Map created with the QGis open source software [98]. (PDF 2240 kb) Additional file 2.
**Results.** Supplementary text for Results section. (PDF 157 kb) Additional file 3: Figure S2. Schematic diagram of the NAT2 870 bp-long single protein-coding Exon (Exon 2) on 8p22. The first (+1) and last positions (+873) of the ORF are indicated on top of it. The positions of the 30 polymorphic sites (SNPs) observed among the 1192 individuals from the 39 African samples analyzed in this study are shown as heavy-black (non-synonymous mutations) or light-gray (synonymous mutations) vertical bars below the diagram. Segments link the SNPs positions to the list of 61 haplotypes inferred from the combination of the 30 SNPs. Haplotypes’ associated acetylation activity (taken from the official NAT2 gene nomenclature, http://nat.mbg.duth.gr/) and average frequency among the 39 population samples are shown on the left and right sides of the list, respectively. (PDF 21 kb) Additional file 4: Table S1.
*NAT2* coding-exon SNPs and haplotypes observed in all sequenced African population samples. (XLSX 32 kb) Additional file 5: Table S2. Diversity of the *NAT2* 870 bp coding-exon in the 39 African populations included in this study. (XLSX 16 kb) Additional file 6: Figure S3. Plot of the number of alleles (distinct *NAT2* sequence haplotypes) observed in samples as a function of sample size. The dashed line shows the linear regression of number of haplotypes on sample size, and Pearson’s product–moment correlation coefficient is shown in the bottom-right caption. (PDF 2 kb) Additional file 7: Figure S4. Frequency distributions of *NAT2* haplotypes in African populations screened for sequence variation in the coding-exon: (a) map showing frequency distributions as pie charts at geographic locations (with size of pie proportional to sample size; map created with the QGis open source software [98]); (b) frequency distributions shown as an area chart, and including the ASW sample of African Americans from the 1000 Genomes Project. Low-activity haplotypes are shown in shades of blue and green, fast haplotypes in shades of red, and those of unknown consequence in shades of gray. (PDF 238 kb) Additional file 8: Figure S5. Variance in *NAT2* haplotype frequencies among African populations. The frequency variance in the complete 39 populations dataset is shown by light-gray bars, that of the AFR dataset (excluding ASW) by dark-gray bars. Haplotypes are ordered by decreasing variance, and only haplotypes displaying variance > 4.83e-4 are shown. (PDF 1 kb) Additional file 9: Figure S6. Graphical representation of the matrix of pairwise Reynolds genetic distances among the 13 populations of the FPLS dataset (left-pane) and of the associated significance (right-pane). (PDF 41 kb) Additional file 10: Figure S7. Plot of Reynolds pairwise genetic distances among the 13 populations of the FPLS dataset as a function of geographic distance separating them (great-circle distances, in km). (PDF 2 kb) Additional file 11: Figure S8. MDS plot of pairwise Reynolds genetic distances between the 29 populations of the FP dataset. The Stress value is 0.045. The same plot is reproduced 4 times, with populations color-coded according to: (a) geographical region, (b) linguistic affiliation, (c) subsistence mode, and (d) biome (see text). (PDF 11 kb) Additional file 12: Figure S9. MDS plot of pairwise Reynolds genetic distances between the 13 populations of the FPLS dataset. The Stress value is 0.014. The same plot is reproduced 4 times, with populations color-coded according to: (a) geographical region, (b) linguistic affiliation, (c) subsistence mode, and (d) biome (see text). (PDF 10 kb) Additional file 13: Table S3. Predicted frequency of slow acetylators per population, with 95% non-parametric (bootstrap) confidence interval. (XLSX 12 kb) Additional file 14: Table S4. Pairwise Wilcoxon rank sum tests of differences in predicted frequency of NAT2 slow acetylation among subsistence modes. (XLSX 11 kb) Additional file 15: Table S5. Single factor and multiple factors ANOVAs of differences in predicted frequency of NAT2 slow acetylation, and Tukey’s HSD tests for multiple comparisons of means. (XLSX 16 kb) Additional file 16: Figure S10 Plot of the number of alleles (distinct *NAT2* sequence haplotypes) of each functional category (green for slow, brown for fast, orange for unknown) observed in samples as a function of sample size. The dashed lines show the linear regression of number of haplotypes on sample size, and Pearson’s product–moment correlation coefficients are provided in the top-left caption. (PDF 2 kb)

## Abbreviations

NAT2: Arylamine N-acetyltransferase 2
ORF: Open reading frame
SNPs: Single nucleotide polymorphisms
bp: Base pairs
AFR: African populations dataset
FP: African food-producing populations dataset
FPLS: African food-producing populations dataset comprising only those samples with size ≥ 20 individuals
AMOVA: Analysis of molecular variance
ANOVA: Analysis of variance
MDS: Nonmetric multidimensional scaling
Geo: Categorization according to geographic location
Lang: Categorization according to linguistic affiliation
Subsist: Categorization according to subsistence mode
Clim: Categorization according to climatic zone and biome
ND: Not defined
mtDNA: Mitochondrial DNA
HLA: Human Leukocyte Antigen
ML: Maximum likelihood
EM: Expectation-maximisation
FDR: False discovery rate
HSD: Honest significant difference
GRCh37/hg19: Human genome reference sequence (Genome Reference Consortium GRCh37, hg19 assembly)
