# Supplementary figures and images for: Variation in NAT2 acetylation phenotypes is associated with differences in food-producing subsistence modes and ecoregions in Africa
Source: BMC Evol Biol. 2015 Dec 1;15:263. doi: 10.1186/s12862-015-0543-6 (PMC4665893; doi:10.1186/s12862-015-0543-6)

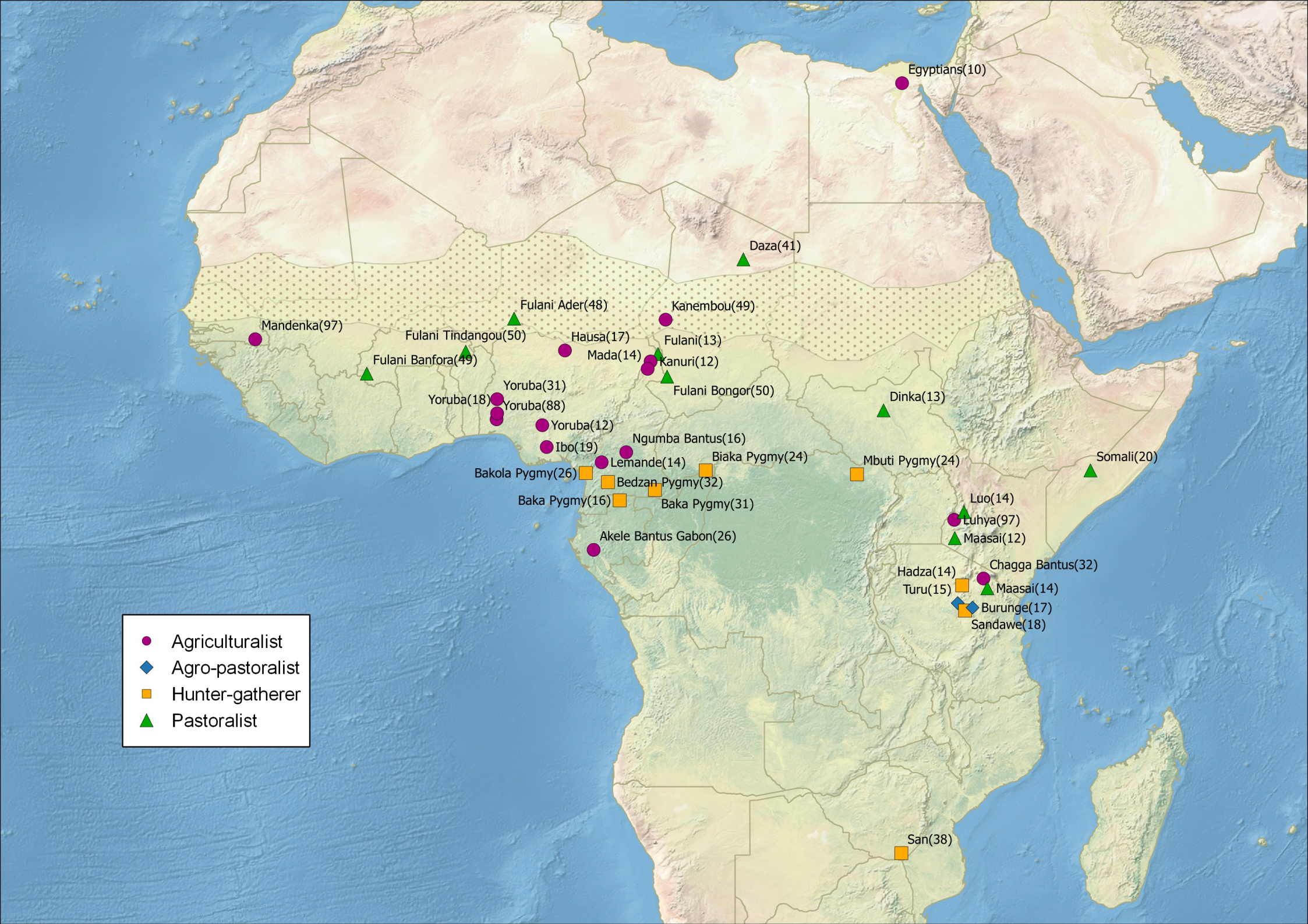

Supplement: Additional file 1: Figure S1. — Map showing the location of African populations screened for sequence variation in NAT2, including the six Sahelian populations of this study. The ASW sample of African Americans from the 1000 Genomes Project is not located on this map. Map created with the QGis open source software [98]. (PDF 2240 kb) [file 12862_2015_543_MOESM1_ESM.pdf]

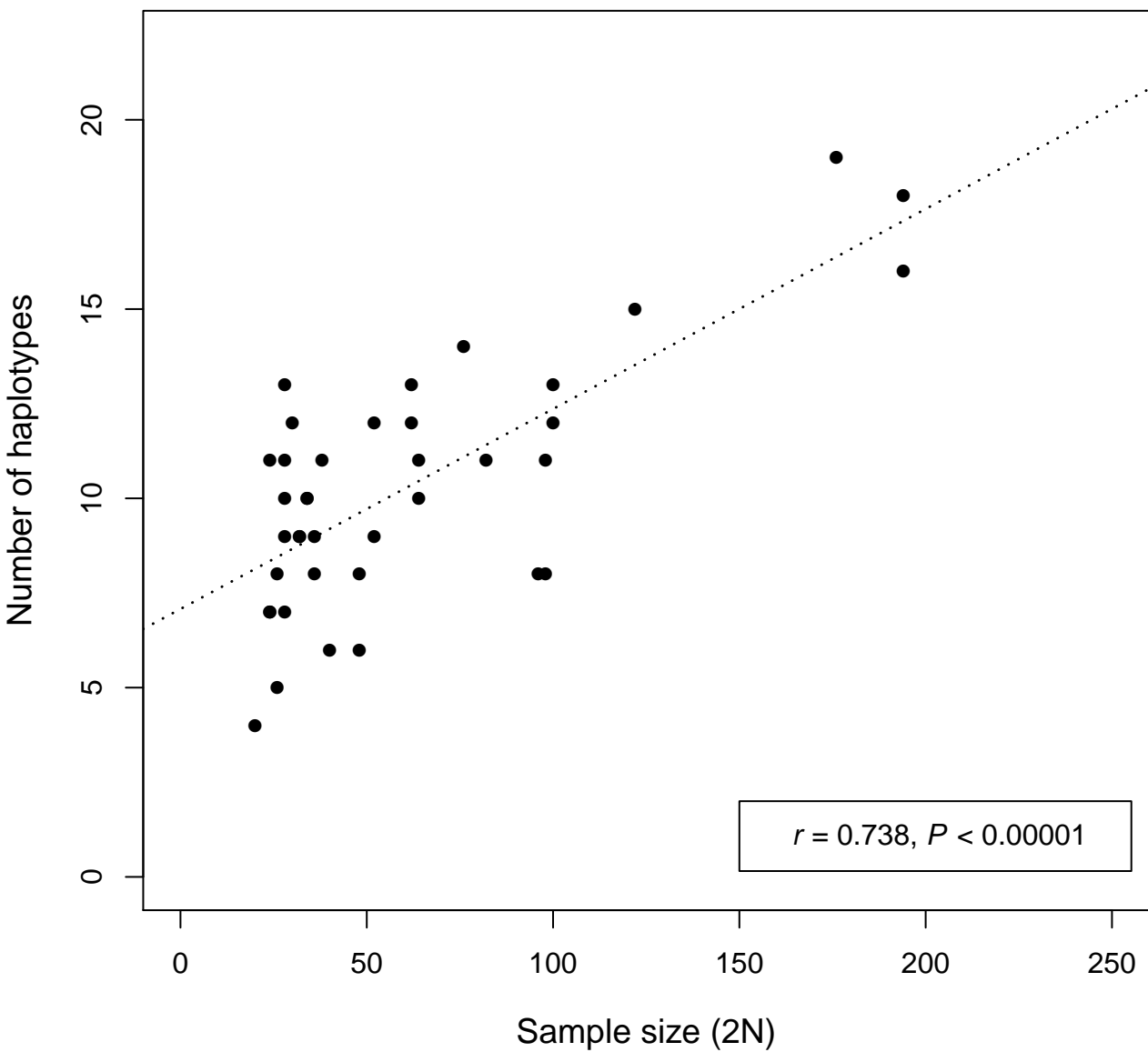

Supplement: Additional file 6: Figure S3. — Plot of the number of alleles (distinct NAT2 sequence haplotypes) observed in samples as a function of sample size. The dashed line shows the linear regression of number of haplotypes on sample size, and Pearson’s product–moment correlation coefficient is shown in the bottom-right caption. (PDF 2 kb) [file 12862_2015_543_MOESM6_ESM.pdf]

a

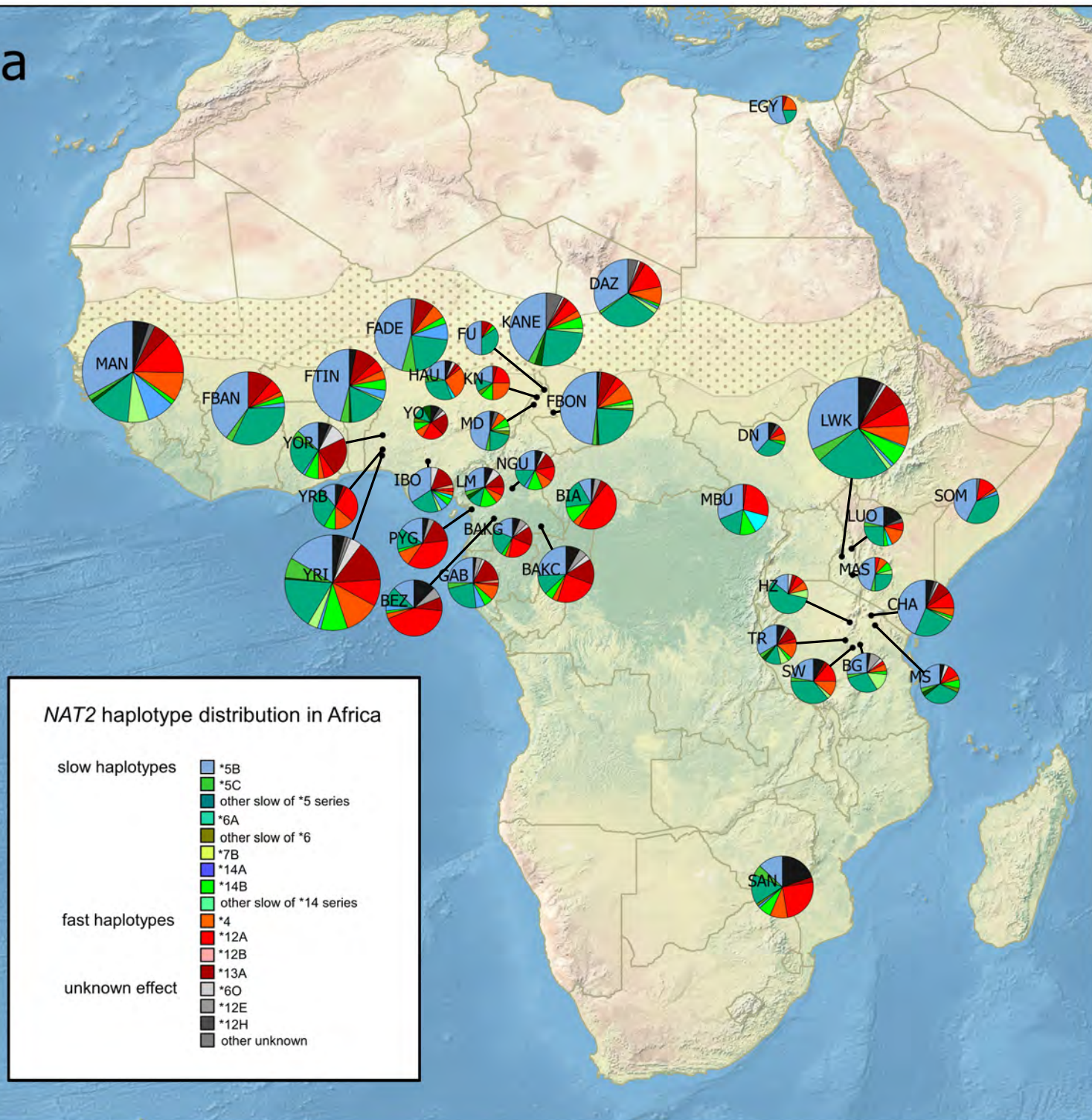

b

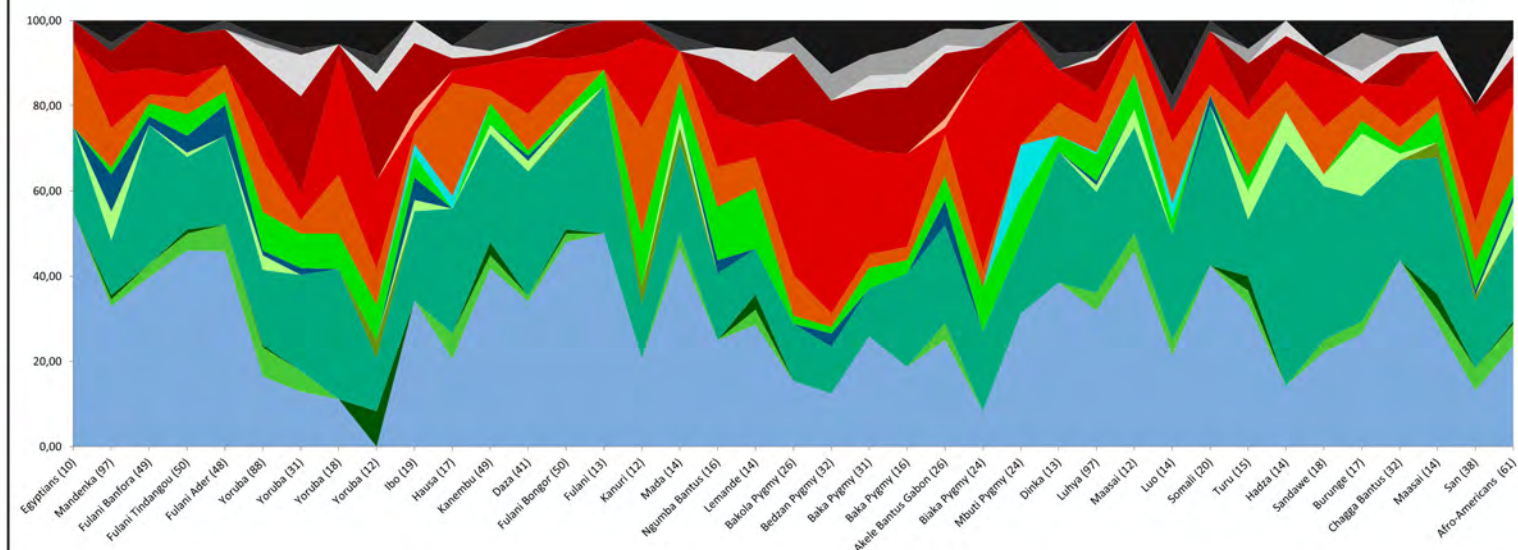

Supplement: Additional file 7: Figure S4. — Frequency distributions of NAT2 haplotypes in African populations screened for sequence variation in the coding-exon: (a) map showing frequency distributions as pie charts at geographic locations (with size of pie proportional to sample size; map created with the QGis open source software [98]); (b) frequency distributions shown as an area chart, and including the ASW sample of African Americans from the 1000 Genomes Project. Low-activity haplotypes are shown in shades of blue and green, fast haplotypes in shades of red, and those of unknown consequence in shades of gray. (PDF 238 kb) [file 12862_2015_543_MOESM7_ESM.pdf]

## Variance of haplotype frequencies among populations

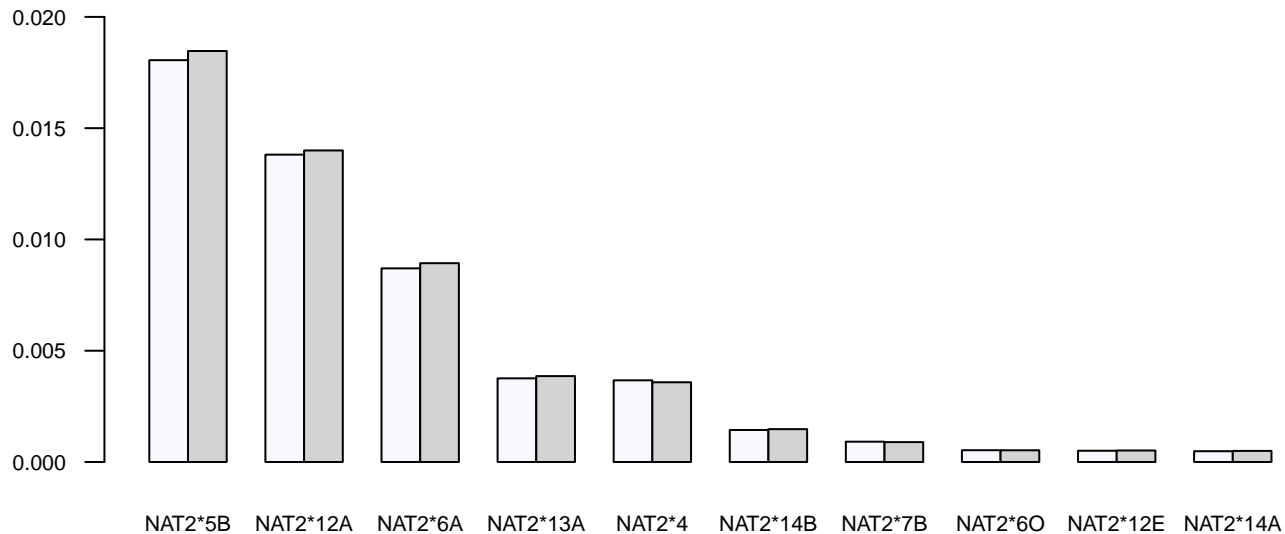

Supplement: Additional file 8: Figure S5. — Variance in NAT2 haplotype frequencies among African populations. The frequency variance in the complete 39 populations dataset is shown by light-gray bars, that of the AFR dataset (excluding ASW) by dark-gray bars. Haplotypes are ordered by decreasing variance, and only haplotypes displaying variance > 4.83e-4 are shown. (PDF 1 kb) [file 12862_2015_543_MOESM8_ESM.pdf]

[illegible]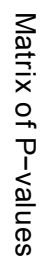

Supplement: Additional file 9: Figure S6. — Graphical representation of the matrix of pairwise Reynolds genetic distances among the 13 populations of the FPLS dataset (left-pane) and of the associated significance (right-pane). (PDF 41 kb) [file 12862_2015_543_MOESM9_ESM.pdf]

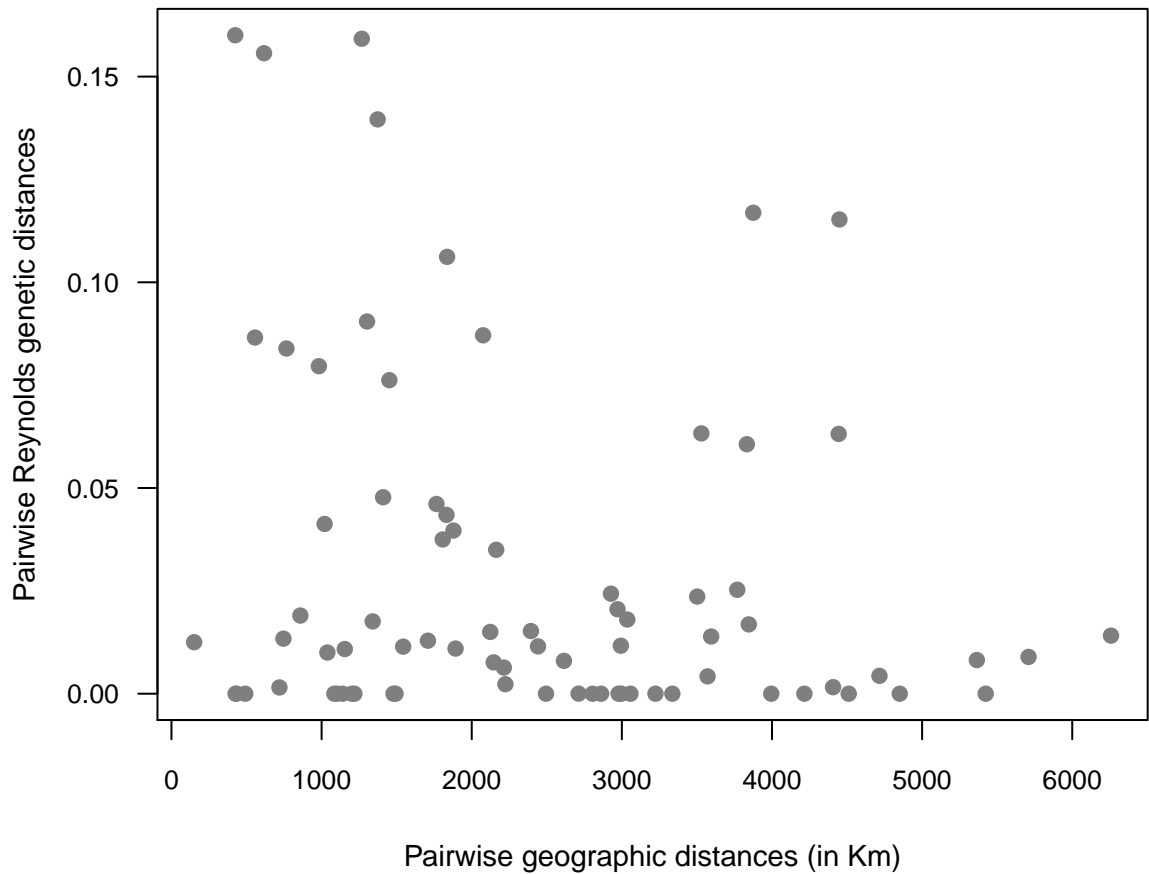

Supplement: Additional file 10: Figure S7. — Plot of Reynolds pairwise genetic distances among the 13 populations of the FPLS dataset as a function of geographic distance separating them (great-circle distances, in km). (PDF 2 kb) [file 12862_2015_543_MOESM10_ESM.pdf]

**Geographic location**

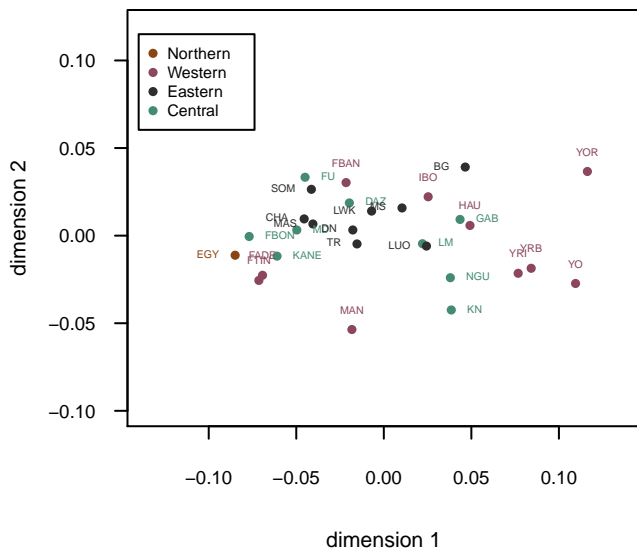

**Linguistic affiliation**

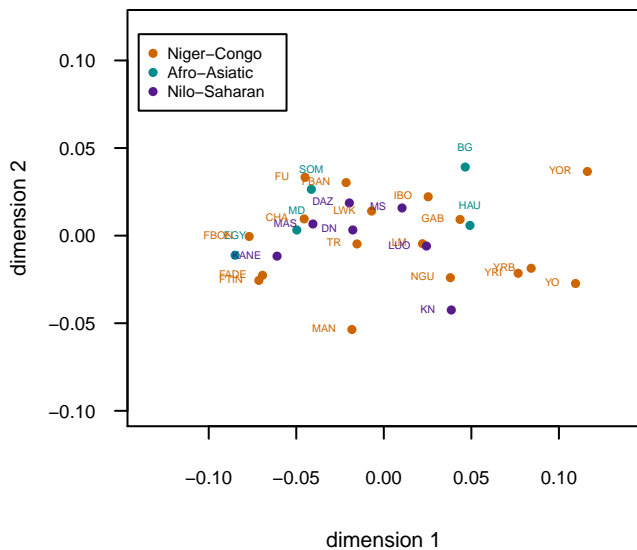

**Subsistence mode**

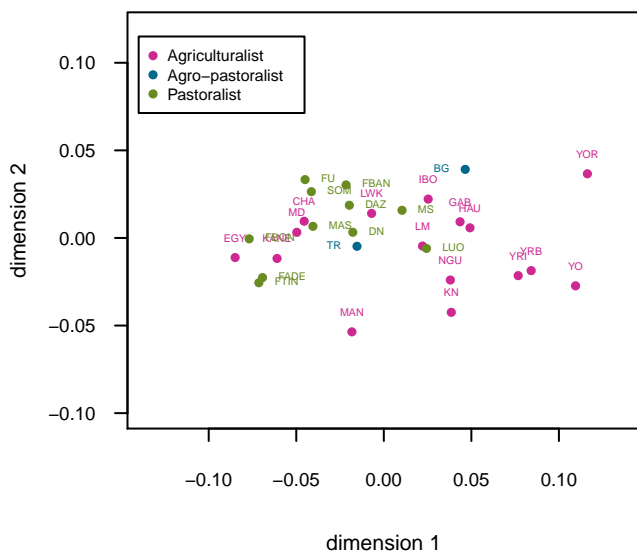

**Biome**

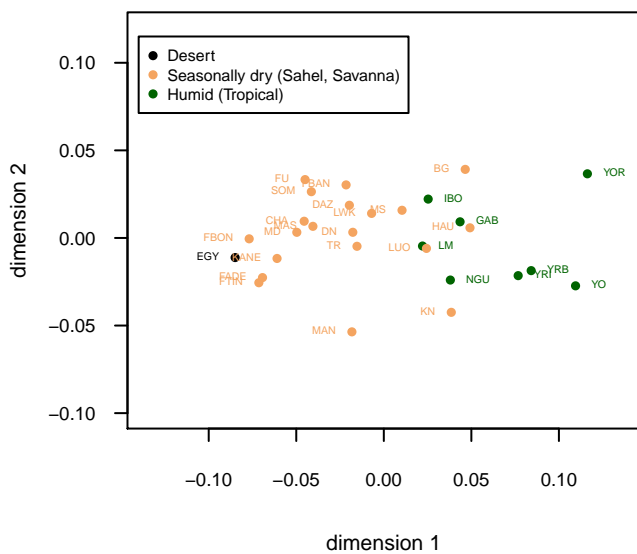

Supplement: Additional file 11: Figure S8. — MDS plot of pairwise Reynolds genetic distances between the 29 populations of the FP dataset. The Stress value is 0.045. The same plot is reproduced 4 times, with populations color-coded according to: (a) geographical region, (b) linguistic affiliation, (c) subsistence mode, and (d) biome (see text). (PDF 11 kb) [file 12862_2015_543_MOESM11_ESM.pdf]

**Geographic location**

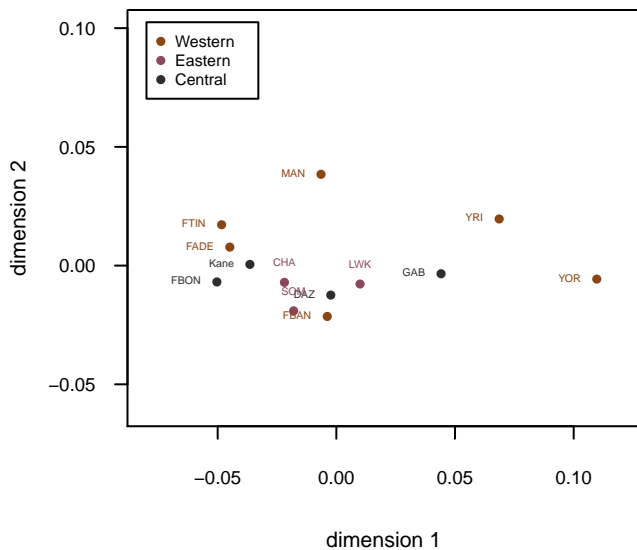

**Linguistic affiliation**

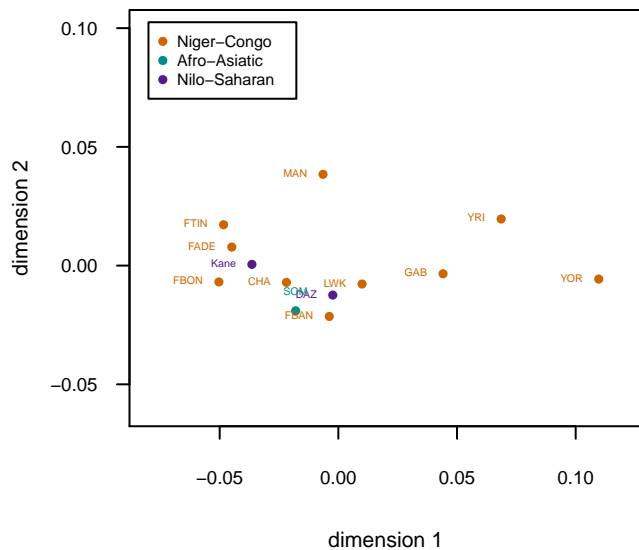

**Subsistence mode**

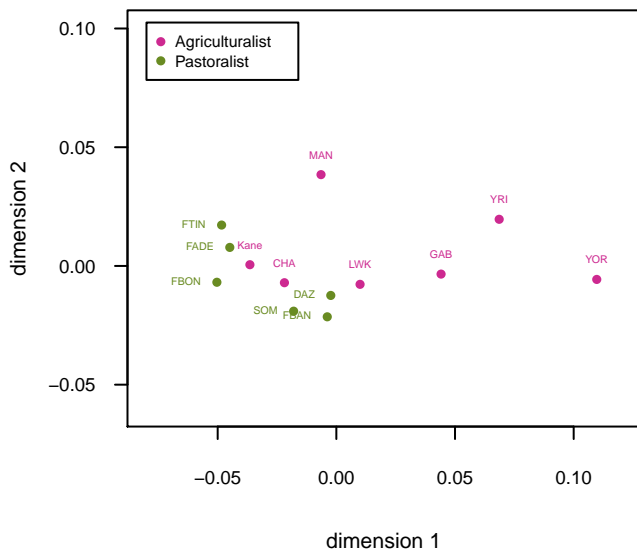

**Biome**

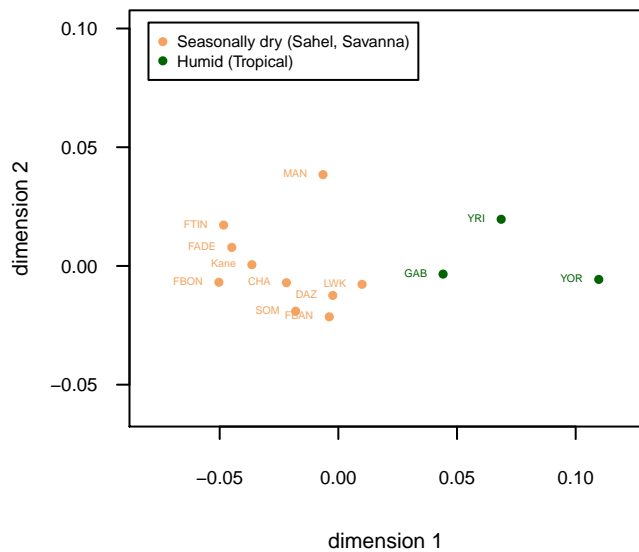

Supplement: Additional file 12: Figure S9. — MDS plot of pairwise Reynolds genetic distances between the 13 populations of the FPLS dataset. The Stress value is 0.014. The same plot is reproduced 4 times, with populations color-coded according to: (a) geographical region, (b) linguistic affiliation, (c) subsistence mode, and (d) biome (see text). (PDF 10 kb) [file 12862_2015_543_MOESM12_ESM.pdf]

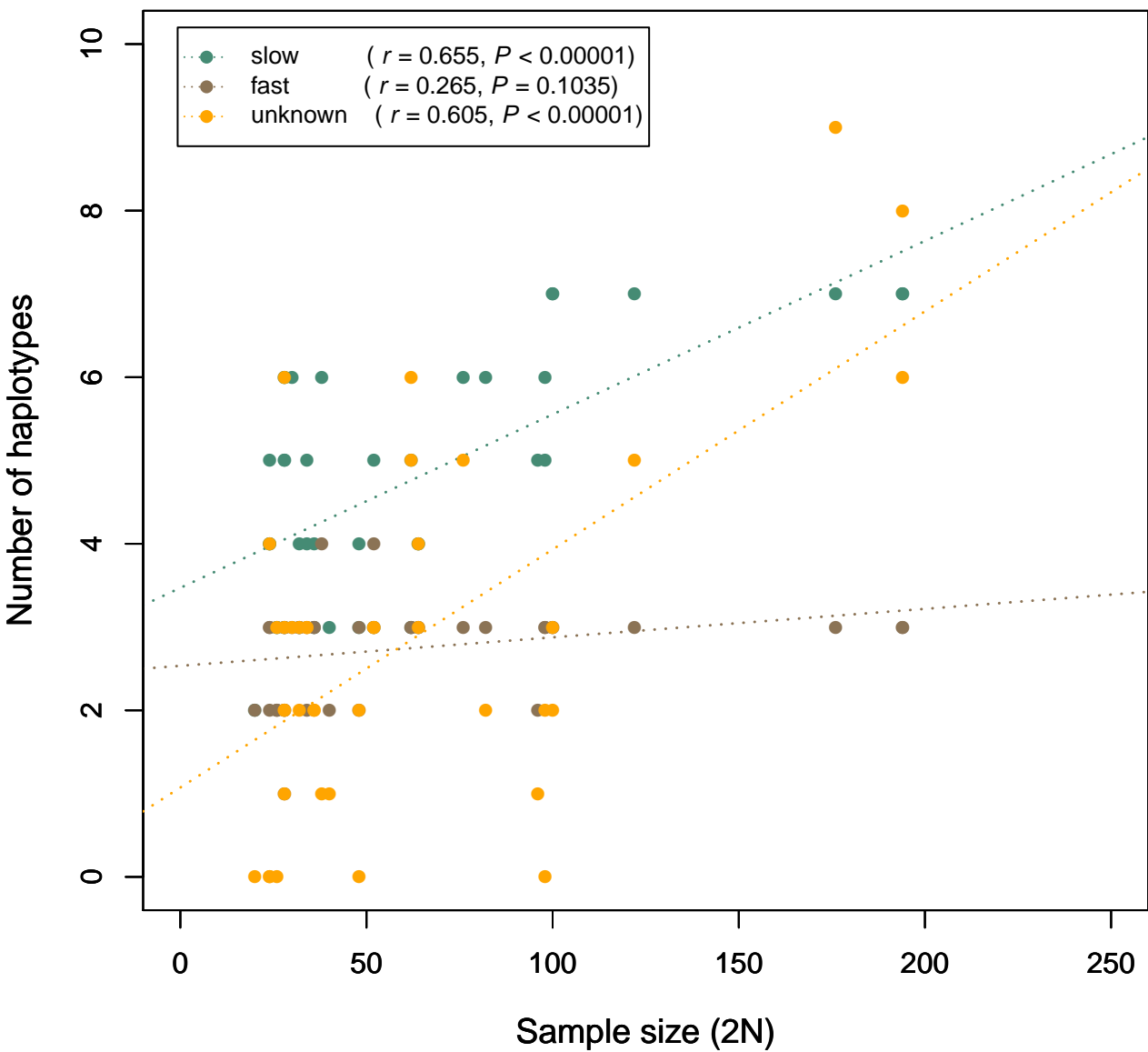

Supplement: Additional file 16: Figure S10 — Plot of the number of alleles (distinct NAT2 sequence haplotypes) of each functional category (green for slow, brown for fast, orange for unknown) observed in samples as a function of sample size. The dashed lines show the linear regression of number of haplotypes on sample size, and Pearson’s product–moment correlation coefficients are provided in the top-left caption. (PDF 2 kb) [file 12862_2015_543_MOESM16_ESM.pdf]
