# Supplementary material for: Variation in NAT2 acetylation phenotypes is associated with differences in food-producing subsistence modes and ecoregions in Africa
Source: BMC Evol Biol. 2015 Dec 1;15:263. doi: 10.1186/s12862-015-0543-6 (PMC4665893; doi:10.1186/s12862-015-0543-6)
Supplement: Additional file 2. — Results. Supplementary text for Results section. (PDF 157 kb) [file 12862_2015_543_MOESM2_ESM.pdf]

Additional Results for:

Variation in NAT2 acetylation phenotypes is associated with differences in food-producing subsistence modes and ecoregions in Africa

Eliška PODGORNÁ, Issa DIALLO, Christelle VANGENOT, Alicia SANCHEZ-MAZAS, Audrey SABBAGH, Viktor ČERNÝ and Estella S. POLONI

NAT2 diversity in the Sahel **p. 2**

Sequencing results p. 2

Phase reconstruction results p. 2

Haplotype frequency distributions results p. 4

NAT2 diversity in African populations **p. 5**

NAT2 haplotypes observed in the 39 African samples p. 5

Correlation between sample size and number of *NAT2* haplotypes detected p. 6

NAT2 population structure in Africa **p. 6**

Proportions of significant pairwise genetic distances in the FPLS dataset p. 6

Association of _NAT2_ genetic structure with geography, culture, or climatic zone and biome **p. 7**

Hierarchical AMOVA results p. 7

MDS analyses of genetic distances among populations p. 8

Additional Results references **p. 9**

NAT2 diversity in the Sahel

Sequencing results

We sequenced 1,396 bp encompassing the 870 bp *NAT2* coding exon in 287 samples from the six Sahelian populations of this study (Fulani from Banfora and from Tindangou in Burkina Faso, Fulani from Ader in Niger, Fulani from Bongor in Chad, and Daza and Kanembou in Chad), i.e. 26 bp upstream, and 500 bp downstream of the exon, respectively.
In total, 15 polymorphic positions were observed, 11 of which are located in the coding exon, and 9 being non-synonymous (see Figure 1 in the main text). Among the latter, a SNP at position 121 in the coding exon was detected in a single Fulani from Tindangou in heterozygous state. This SNP, which was confirmed through both forward and reverse sequencing with two partially overlapping pairs of primers (see Methods in the main text), was not yet included in the official *NAT2* nomenclature but already listed in Ensembl (rs149283608, see below).

Phase reconstruction results

As explained in the main text (Methods), we used two approaches to infer the sequence haplotypes and their associated maximum likelihood (ML) frequencies in each population sample, i.e. the Bayesian approach based on an approximate coalescent model implemented in the software PHASE v.2.1 [1, 2], and the maximum likelihood (ML) approach based on the expectation-maximisation (EM) algorithm implemented in Arlequin ver. 3.5 [3], and compared the resulting inferences.
For each population sample, multiple instances of four PHASE runs with different seed (-S option) were performed, varying the number of iterations of the estimation procedure and varying the frequency threshold for a haplotype to be called (-F option), so as to define optimum settings for most parsimonious haplotype inference. A similar approach was adopted for exploring the outputs of the EM algorithm implemented in Arlequin, varying the number of iterations of the E- and M-steps; here, variable values of the convergence criterion (convergence is achieved when the change in log-likelihood between two

successive iterations is smaller than a given epsilon value) were explored to define the optimum settings for most parsimonious haplotype inference. The two softwares used to generate phased haplotypes in the six population samples leaded to almost fully concordant results with the following settings: in PHASE, the number of iterations of the estimation procedure was increased to 10,000, and the -F option was also increased to 0.5 (meaning that a haplotype will be called if even a single individual is carrying it with a 0.5 % probability); in Arlequin, the number of iterations of the E- and M-steps was set to 10,000, and the convergence criterion was set to the minimum possible value (epsilon value = 1e-12). The only exception to the full concordance of results obtained with the two softwares was a single Daza individual for which PHASE and Arlequin outputs each consisted of a different single diploid haplotype combination in which the rare haplotype called was different. Because we observed that PHASE results were less stable among runs (slightly different haplotype calls output with different seed numbers but with the same settings) than Arlequin results (identical haplotype calls with the same settings), we chose the latter software to obtain the haplotype calls and their associated ML frequencies. We thus adopted the Arlequin output for this single Daza individual.

Thus, phase reconstruction with the EM algorithm of Arlequin was obtained unambiguously (i.e. a single combination of two haplotypes with an associated probability of 1.0) in 100% of the Daza and Fulani from Banfora samples, and in ca. 95% of the Kanembou, Fulani from Ader and Fulani from Tindangou. In the Fulani from Bongor, unique diploid haplotype combinations were only obtained for 76% of the individuals. However, for all individuals for whom an ambiguous phase reconstruction was obtained, only two diploid haplotype combinations were output per genotype, one with an associated probability higher than 99% and the other being under 1%. Note that identical results were obtained with the Bayesian approach of PHASE, except for a single Daza individual (as explained above).

Twenty-one haplotypes were thus parsimoniously inferred for the six Sahelian samples, of which three haplotypes not yet described to the best of our knowledge. One of them, haplotype *NAT2*12N*, bears the 803A>G non-synonymous mutation that defines the *NAT2*12* allelic series, in combination

with the newly observed 121A>T transversion (Ensembl rs149283608). The other two, *NAT2*13D* and *NAT2*14K*, are defined by a new combination of recognized signature SNPs of the *NAT2*13* and *NAT2*14* allelic series, respectively, with other known SNPs (haplotype *NAT2*13D* has the 282C>T synonymous mutation that defines *NAT2*13* alleles together with the non-synonymous transition 766A>G, and *NAT2*14K* has both the 191G>A signature SNP of *NAT2*14* alleles and the 282C>T synonymous mutation of *NAT2*14B, NAT2*14D, NAT2*14G*, *NAT2*14H* and *NAT2*14J*, in addition to non-synonymous transition 838G>A). While *NAT2*12N* and *NAT2*13D* were each observed only once in a single Fulani heterozygote (from the Tindangou and Bongor areas, respectively), *NAT2*14K* was observed in a Fulani from Tindangou and a Luo individual, i.e. an individual from a Nilo-Saharan pastoralist population in Kenya (Additional Table 1). All three were a priori considered as "unknown effect" alleles with respect to enzymatic activity.

Haplotype frequency distributions

Slow haplotypes. The most frequent haplotype in the six Sahelian populations is low-activity haplotype *NAT2*5B* (Figure 1 and Table 2), with an average frequency among population samples of 41.9% (i.e. 42.9% of the total sample, frequencies in individual samples varying from 34.1% in the Daza to 48% in the Fulani from Bongor, including *NAT2*5Ba*).

The next most common haplotype is *NAT2*6A*, also a low-activity haplotype, with an average frequency among population samples of 24.5% (24.6% of the total sample, varying from 17% in the Fulani from Tindangou to 32.7% in the Fulani from Banfora, including *NAT2*6Aa*).

Together, these two slow haplotypes thus account for 67.4% of the gene copies in the total sample of our study (from 63% in the Fulani from Tindangou to 72.4% in the Fulani from Banfora). Further low-activity haplotypes, averaging 11.3%, include *NAT2*5C*, **14A*, and **14B* with frequencies ranging from 1.2% to 7.3%, and **5A*, **7B* and *6C*, from 1% to 3.1%.

Fast haplotypes. Only three different fast haplotypes were detected in the Sahelian samples (*NAT2*4*, *NAT2*12A* and *NAT2*13A*), and these represent on

average less than 18% of the gene copies. While haplotype *NAT2*13A* is commonly found in the Fulani nomads (between 7% and 11.2%), its frequency is lower in the sedentary Kanembou and semi-nomadic Daza (2% and 2.4%, respectively). In those latter samples, haplotype *NAT2*12A* is observed at frequencies of 6.1% and 13.4%, respectively, whereas in the Fulani its highest frequency is observed among those from Banfora (6.1%). Finally, the frequencies of *NAT2*4*, the haplotype classically considered as the "wild type", range from 2% in the Fulani from Banfora to 8.5% in the Daza.

Unknown activity haplotypes. Six haplotypes detected in the Sahelian samples could not be classified according to their effect on acetylation status. These include the the newly inferred haplotypes *NAT2*12N*, *NAT2*13D*, and *NAT2*14K*, plus three haplotypes whose acetylation status is reported as unknown in the official *NAT2* gene nomenclature (nat.mbg.duth.gr/), namely *NAT2*6F*, *NAT2*6O*, and *NAT2*12H*. Their total frequency is of 3% or less in the Fulani samples, whereas it is of 8.2% and 6.1% in the Kanembou and Daza, respectively.

NAT2 diversity in African populations

NAT2 haplotypes observed in the 39 African samples

Sixty-one *NAT2* haplotypes were detected in the coding-exon sequences of 39 African samples (1,192 individuals, Additional Figure 2 and Additional Table 1), of which 20 newly described haplotypes, 3 of which in the Sahelian samples of this study (see above). Only two of these newly inferred haplotypes were observed more than once: haplotype *NAT2*14K* was detected in two heterozygous individuals from distinct pastoralist populations (one Niger-Congo Fulani from Tindangou and one Nilo-Saharan Luo from Kenya), whereas 10 counts of haplotype *NAT2*26* (always in heterozygous state) were inferred in the San sample from Zimbabwe. The remaining eighteen new haplotypes were observed each only once in a single heterozygous individual.

Correlation between sample size and number of *NAT2* haplotypes detected

As explained in the main text, we found a high and significant correlation between sample size and number of haplotypes detected ($r = 0.738$, $P < 0.00001$, Additional Figure 3). This correlation remains significant even after removal of the four samples with sizes larger than 50 individuals, namely after removal of the Yoruba (YRI), Luhya (LWK) and African Americans (ASW) from the 1000 Genomes Project [4] and of the Mandenka from [5] ($r = 0.398$, $P = 0.018$). Because we observe that the number of slow haplotypes detected is higher than that of fast ones in most populations, the possibility exists that, due to small size sampling, predicted slow phenotype's prevalence is often underestimated. The results shown in Additional Figure 10, which report a significant positive correlation of sample size with number of haplotypes detected for slow-causing variants ($r = 0.655$, $P < 0.00001$) but not for fast ones ($r = 0.265$, $P = 0.1035$), bring support to this idea. However, this hypothesis is challenged by the detection of high numbers of haplotypes with unknown effect, although generally at low frequencies, but nevertheless also correlated with sample size ($r = 0.605$, $P < 0.00001$). Testing this hypothesis needs thus to await for future studies that will uncover the acetylation status associated with haplotypes of unknown effect.

NAT2 population structure in Africa

Proportions of significant pairwise genetic distances in the FPLS dataset

In the FPLS dataset, 34.6% of the pairwise genetic distances between populations were found significant. As indicated in the main text, most of these significant distances differentiate the Yoruba and Akele populations from the others (Additional Figure 6): the two Yoruba samples (YRI and YOR) were found significantly differentiated from all other populations but the Gabonese Akele Bantus (GAB), which in turn were found differentiated from three Fulani groups (FTIN, FADE and FBON) and from the Kanembou; the Fulani from Tindangou also differentiated from the Kenyan Luhya (LWK), whereas the Mandenka

differentiated from two other Fulani groups (FBAN and FBON). In turn, among the six Sahelian populations, none of the estimated genetic distances was found significant, and most of the genetic distances between Sahelian and East African populations were also found statistically not significant.

Association of *NAT2* genetic structure with geography, culture, or climatic zone and biome

Hierarchical AMOVA results

The results reported in Figure 3 indicate that neither differentiation among geographic groups nor among linguistic groups does associate with the genetic structure of populations displayed by *NAT2* sequences (Φ_{CT} indices not significant for any of the AFR, FP, or FPLS datasets). Conversely, significant Φ_{CT} indices were observed with a classification according to subsistence strategy in the AFR and FP datasets (1.8% and 1.2%, respectively, *P*-values smaller than 5%, and remaining significant only for AFR after Bonferroni correction for type I error risk), although not with that of the FPLS dataset. In any case, under this subsistence mode categorization criterion, the Φ_{CT} index is lower than the Φ_{SC} index (Φ_{SC} from 1.6% to 2.1 %, $P < 0.0001$), thereby meaning that more differentiation is found among populations in groups with matching subsistence mode than between those groups, even if only slightly more so. In turn, as explained in the main text, under the last hierarchical analysis scheme, which corresponds to a classification according to ecoregion, Φ_{CT} indices were found high and significant for all three data subsets (Φ_{CT} of 2.3%, 3.6% and 5.4%, for AFR, FP, and FPLS, respectively, all *P*-values < 0.05 and remaining significant after Bonferroni correction for multiple testing) and greater than Φ_{SC} indices in all cases (corresponding Φ_{SC} for AFR, FP and FPLS of 2.2%, 0.7% and 0.6%, respectively, all *P*-values < 0.05).

MDS analyses of genetic distances among populations

Consistent with the AMOVA results, categorization according to the environment seems to better fit with the location of populations in the MDS plots than any of the three other criteria. Indeed, for the AFR dataset (Figure 1), the MDS plot displays populations in a scattered fashion, with no obvious clusters, and with most hunter-gatherer populations located in peripheral positions. A scattered pattern of differentiation without clear clustering of the populations is also observed in the MDS plot of the FP dataset (Additional Figure 8). Here, the most differentiated populations are the Yoruba and a few other populations living in the humid tropical zones around the Gulf of Guinea, while the other populations both from the Sahelian zone and from East Africa tend to display smaller genetic differentiation between them. The latter observation is further supported by the MDS plot of the FPLS dataset (Additional Figure 9). Here, the Yoruba and Akele Bantus from Gabon, and to a lesser extent the Mandenka, differentiate from all others. For each of the three datasets, we observe that the highlighting of the populations according to our four categorization criteria suggests a stronger association of *NAT2* genetic structure with a differentiation of populations according to the environment than to lifestyle, in agreement with the hierarchical AMOVA results. For the FP and FPLS datasets in particular, populations with distinct subsistence mode tend to be located in distinct areas of the MDS plots, but those areas do also substantially overlap, mainly because the locations of agriculturalists appear as more scattered in the plot. Conversely, populations tend to be located in distinct areas of the MDS plots depending on whether they live in the seasonally dry zones (Sahel, Savanna) or to the south and west of it, i.e. in the humid tropical and equatorial zones (Additional Figures 8 and 9).

Additional Results references

1. Stephens M, Scheet P: **Accounting for decay of linkage disequilibrium in haplotype inference and missing-data imputation**. *American journal of human genetics* 2005, **76**(3):449-462.
2. Stephens M, Smith NJ, Donnelly P: **A new statistical method for haplotype reconstruction from population data**. *American journal of human genetics* 2001, **68**(4):978-989.
3. Excoffier L, Lischer HE: **Arlequin suite ver 3.5: a new series of programs to perform population genetics analyses under Linux and Windows**. *Molecular ecology resources* 2010, **10**(3):564-567.
4. Genomes Project C, Abecasis GR, Auton A, Brooks LD, DePristo MA, Durbin RM, Handsaker RE, Kang HM, Marth GT, McVean GA: **An integrated map of genetic variation from 1,092 human genomes**. *Nature* 2012, **491**(7422):56-65.
5. Sabbagh A, Langaney A, Darlu P, Gerard N, Krishnamoorthy R, Poloni ES: **Worldwide distribution of NAT2 diversity: implications for NAT2 evolutionary history**. *BMC genetics* 2008, **9**:21.
