# Supplementary material for: Variation in NAT2 acetylation phenotypes is associated with differences in food-producing subsistence modes and ecoregions in Africa
Source: BMC Evol Biol. 2015 Dec 1;15:263. doi: 10.1186/s12862-015-0543-6 (PMC4665893; doi:10.1186/s12862-015-0543-6)
Supplement: Additional file 3: Figure S2. — Schematic diagram of the NAT2 870 bp-long single protein-coding Exon (Exon 2) on 8p22. The first (+1) and last positions (+873) of the ORF are indicated on top of it. The positions of the 30 polymorphic sites (SNPs) observed among the 1192 individuals from the 39 African samples analyzed in this study are shown as heavy-black (non-synonymous mutations) or light-gray (synonymous mutations) vertical bars below the diagram. Segments link the SNPs positions to the list of 61 haplotypes inferred from the combination of the 30 SNPs. Haplotypes’ associated acetylation activity (taken from the official NAT2 gene nomenclature, http://nat.mbg.duth.gr/) and average frequency among the 39 population samples are shown on the left and right sides of the list, respectively. (PDF 21 kb) [file 12862_2015_543_MOESM3_ESM.pdf]

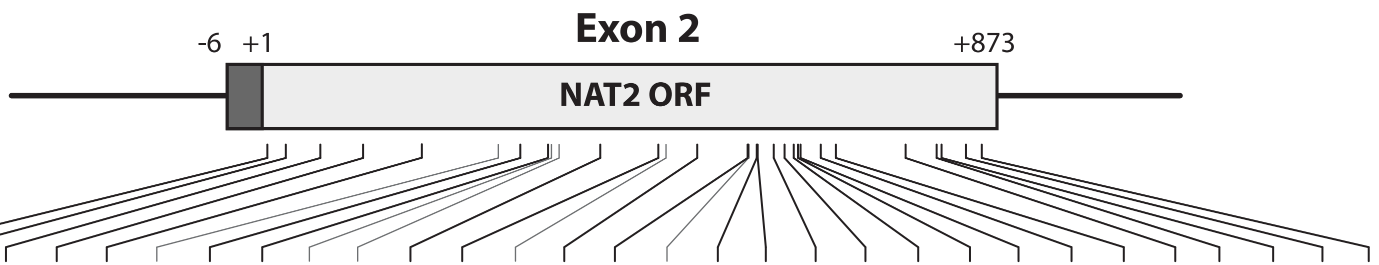

Haplotype	Acetylation activity	A7G	T29C	T70A	A121T	G191A	C282T	C308T	T341C	C345T	T354C	C403G	A472C	C481T	A518G	C578T	G579T	C589T	G590A	G609T	T622C	G633A	C638T	C641T	T665G	C683T	A766G	A803G	T809C	G838A	G857A	Average frequency
Amino acid change		I3V	I10T	L24I	N41Y	R64Q	-	T103I	I114T	-	-	L135V	I158L	-	K173R	T193M	-	R197Stop	R197Q	E203D	Y208H	T211T	P213L	T214I	F23C	P228L	K256E	K268R	I270T	V280M	G286E	
NAT2*4	fast																															8.21
NAT2*12A	fast																											G				12.27
NAT2*12B	fast						T																					G				0.12
NAT2*13A	fast						T																									7.22
NAT2*5A	slow								C					T																		0.66
NAT2*5B	slow								C					T														G				29.04
NAT2*5C	slow								C																			G				2.16
NAT2*5D	slow								C																							0.01
NAT2*6A	slow						T												A													23.01
NAT2*6B	slow																		A													0.03
NAT2*6C	slow						T												A									G				0.42
NAT2*7B	slow						T																								A	1.75
NAT2*14A	slow					A																										1.28
NAT2*14B	slow					A	T																									5.05
NAT2*14D	slow					A	T												A													0.07
NAT2*14E	slow					A																						G				0.43
NAT2*14G	slow					A	T																					G				0.08
NAT2*5L	unknown			A					C					T														G				0.12
NAT2*5M	unknown								C					T														G		A		0.01
NAT2*5N	unknown								C				C	T														G				0.09
NAT2*5KA	unknown						T		C																						A	0.03
NAT2*5TA	unknown						T		C																			G			A	0.08
NAT2*5VA	unknown						T		C					T																	A	0.07
NAT2*5W	unknown								C		C			T														G				0.02
NAT2*5X	unknown								C			G		T														G				0.01
NAT2*5Y	unknown								C					T							C							G				0.01
NAT2*5Z	unknown					A			C					T																		0.03
NAT2*6F	unknown																		A									G				0.07
NAT2*6G	unknown						T								G				A													0.33
NAT2*6H	unknown						T												A								G					0.34
NAT2*6J	unknown						T												A												A	0.01
NAT2*6K	unknown						T												A				T									0.03
NAT2*6L	unknown						T			T									A													0.07
NAT2*6O	unknown						T												A											A		1.63
NAT2*6P	unknown											G							A													0.44
NAT2*6Q	unknown						T	T											A													0.10
NAT2*6U	unknown						T										T		A													0.01
NAT2*6V	unknown											G							A											A		0.07
NAT2*7C	unknown						T																					G			A	0.18
NAT2*12E	unknown						T									T												G				1.09
NAT2*12F	unknown																				C							G				0.21
NAT2*12G	unknown																			T								G				0.38
NAT2*12H	unknown											G																G				1.03
NAT2*12L	unknown																								G			G				0.10
NAT2*12N	unknown				T																							G				0.03
NAT2*12O	unknown		C																	T								G				0.02
NAT2*12P	unknown												C	T														G				0.01
NAT2*13B	unknown						T									T																0.04
NAT2*13D	unknown						T																				G					0.03
NAT2*13E	unknown						T																	T								0.09
NAT2*13F	unknown						T																							A		0.08
NAT2*13G	unknown						T						C																			0.04
NAT2*14H	unknown					A	T																			T						0.24
NAT2*14J	unknown					A	T															A										0.09
NAT2*14K	unknown					A	T																							A		0.12
NAT2*14L	unknown	G				A	T																									0.01
NAT2*22	unknown																			T												0.20
NAT2*23	unknown			A																												0.16
NAT2*24	unknown											G																				0.11
NAT2*26	unknown																												C			0.34
NAT2*27	unknown																	T														0.03
